# Mechanical force induces macrophage-derived exosomal UCHL3 promoting bone marrow mesenchymal stem cell osteogenesis by targeting SMAD1

**DOI:** 10.1186/s12951-023-01836-z

**Published:** 2023-03-14

**Authors:** Panjun Pu, Shengnan Wu, Kejia Zhang, Hao Xu, Jiani Guan, Zhichun Jin, Wen Sun, Hanwen Zhang, Bin Yan

**Affiliations:** 1 Department of Orthodontics, The Affiliated Stomatological Hospital of Nanjing Medical University, Nanjing, 210000 China; 2 Jiangsu Key Laboratory of Oral Diseases, Nanjing Medical University, Nanjing, 210000 China; 3 Jiangsu Province Engineering Research Center of Stomatological Translational Medicine, Nanjing, 210000 China; 4 School of Basic Medical Sciences, Nanjing Medical University, Nanjing, 210000 China; 5 Key Laboratory of Targeted Intervention of Cardiovascular Disease, Collaborative Innovation Center for Cardiovascular Disease Translational Medicine, Nanjing Medical University, Nanjing, 210000 China; 6 Key Laboratory of Shaanxi Province for Craniofacial Precision Medicine Research, College of Stomatology, Xi’an Jiaotong University, Xi’an, 710049 China

**Keywords:** Macrophages, Orthodontic tooth movement, Osteogenesis, Mechanical force, Exosomes, UCHL3, SMAD1

## Abstract

**Background:**

Orthodontic tooth movement (OTM), a process of alveolar bone remodelling, is induced by mechanical force and regulated by local inflammation. Bone marrow-derived mesenchymal stem cells (BMSCs) play a fundamental role in osteogenesis during OTM. Macrophages are mechanosensitive cells that can regulate local inflammatory microenvironment and promote BMSCs osteogenesis by secreting diverse mediators. However, whether and how mechanical force regulates osteogenesis during OTM via macrophage-derived exosomes remains elusive.

**Results:**

Mechanical stimulation (MS) promoted bone marrow-derived macrophage (BMDM)-mediated BMSCs osteogenesis. Importantly, when exosomes from mechanically stimulated BMDMs (MS-BMDM-EXOs) were blocked, the pro-osteogenic effect was suppressed. Additionally, compared with exosomes derived from BMDMs (BMDM-EXOs), MS-BMDM-EXOs exhibited a stronger ability to enhance BMSCs osteogenesis. At in vivo, mechanical force-induced alveolar bone formation was impaired during OTM when exosomes were blocked, and MS-BMDM-EXOs were more effective in promoting alveolar bone formation than BMDM-EXOs. Further proteomic analysis revealed that ubiquitin carboxyl-terminal hydrolase isozyme L3 (UCHL3) was enriched in MS-BMDM-EXOs compared with BMDM-EXOs. We went on to show that BMSCs osteogenesis and mechanical force-induced bone formation were impaired when UCHL3 was inhibited. Furthermore, mothers against decapentaplegic homologue 1 (SMAD1) was identified as the target protein of UCHL3. At the mechanistic level, we showed that SMAD1 interacted with UCHL3 in BMSCs and was downregulated when UCHL3 was suppressed. Consistently, overexpression of SMAD1 rescued the adverse effect of inhibiting UCHL3 on BMSCs osteogenesis.

**Conclusions:**

This study suggests that mechanical force-induced macrophage-derived exosomal UCHL3 promotes BMSCs osteogenesis by targeting SMAD1, thereby promoting alveolar bone formation during OTM.

**Graphical Abstract:**

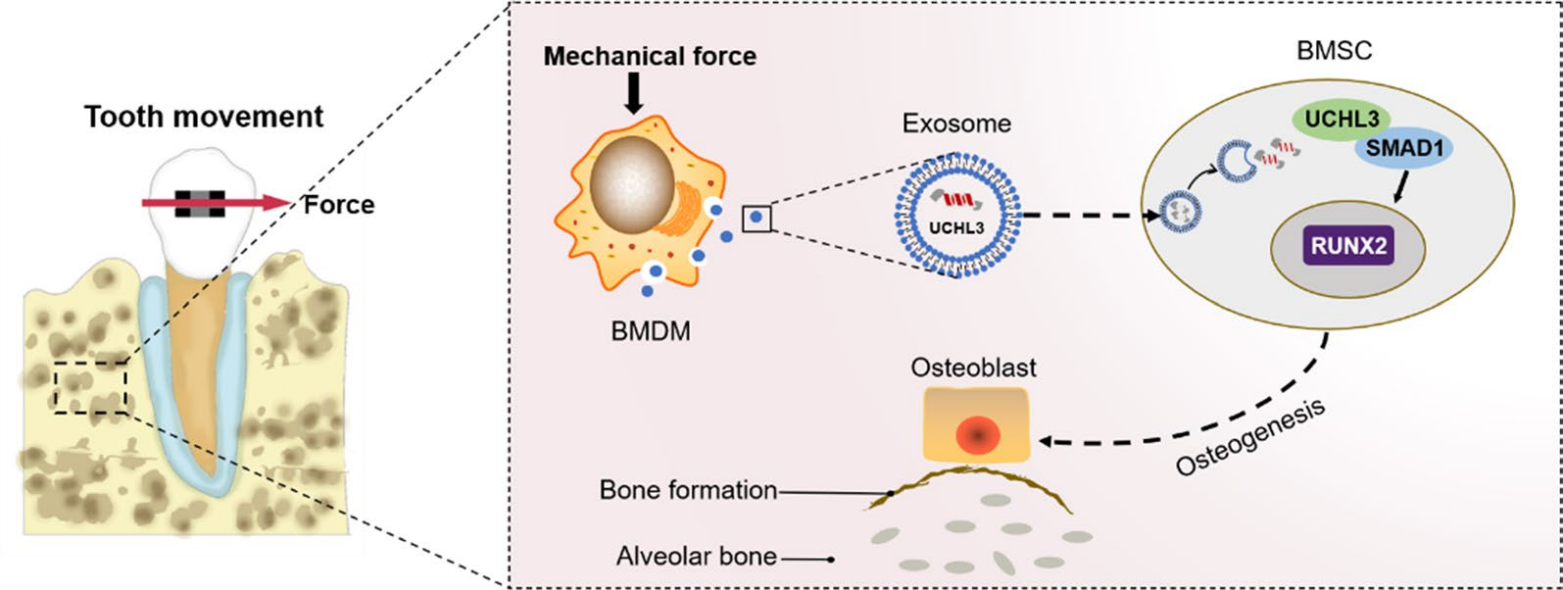

**Supplementary Information:**

The online version contains supplementary material available at 10.1186/s12951-023-01836-z.

## Background

Malocclusion refers to irregular teeth or abnormal occlusion, with a high prevalence ranging from 20 to 100% reported by different researchers [1–3]. Orthodontic treatment is an effective way to correct malocclusion. It is based on the principle that tooth movement occurs when the tooth and its periodontal tissues, such as alveolar bone, are subjected to long-term mechanical force. In essence, orthodontic tooth movement (OTM) is a process of alveolar bone remodelling induced by mechanical force and regulated by local aseptic inflammation, whose underlying mechanism is critical to dissect [4, 5].

In the force-induced alveolar bone remodelling microenvironment, multiple types of cells sense mechanical stimulation and regulate bone remodelling, including macrophages, bone marrow-derived mesenchymal stem cells (BMSCs), periodontal ligament stem cells (PDLSCs), osteoblasts, osteoclasts and osteocytes [6–10]. Macrophages, as mechanosensitive cells [11–13], play a vital role in OTM by secreting cytokines and modulating local inflammation [5, 14, 15]. Previous studies have shown that macrophages are modulated by mechanical force to regulate tooth movement, whereas the mechanism by which macrophages regulate alveolar bone remodelling, especially alveolar bone formation during OTM, is still not clear.

It has been demonstrated that BMSCs, progenitors of osteoblasts, can directly respond to mechanical forces and promote alveolar bone formation during OTM [6]. Macrophages are important modulators of BMSCs by secreting a variety of inflammatory cytokines [16, 17]. These cytokines can regulate the behaviors of BMSCs, including homing, proliferation, and osteogenic differentiation, and further promote bone regeneration [18–20]. Therefore, macrophages modulating BMSCs osteogenesis under mechanical force may be an important component of mechanical force-induced alveolar bone formation during OTM.

Recently, cumulative evidence suggests that exosomes mediate the communication between macrophages and BMSCs in the bone remodelling microenvironment [21, 22]. The bilayer membrane structure of exosomes can protect substances such as nucleic acids, proteins and lipids within it [23]. In addition, exosomes can be directly endocytosed by BMSCs, instead of being inhibited by certain feedback mechanisms like cytokines [24]. However, whether and how exosomes derived from macrophages under mechanical force regulate BMSCs osteogenesis as well as alveolar bone formation remains unknown. Lack of such knowledge is an important problem, since, without it, acquiring the ability to modulate key signaling processes pharmacologically in OTM and malocclusion treatment is highly unlikely.

In this study, we show that mechanical force modulates bone marrow-derived macrophage (BMDM)-derived exosomal ubiquitin carboxyl-terminal hydrolase isozyme L3 (UCHL3), which promotes BMSCs osteogenesis by targeting mothers against decapentaplegic homologue 1 (SMAD1), thereby promoting alveolar bone formation during OTM.

## Results

### Mechanical force promotes BMDM-mediated BMSCs osteogenesis

**Fig. 1:**
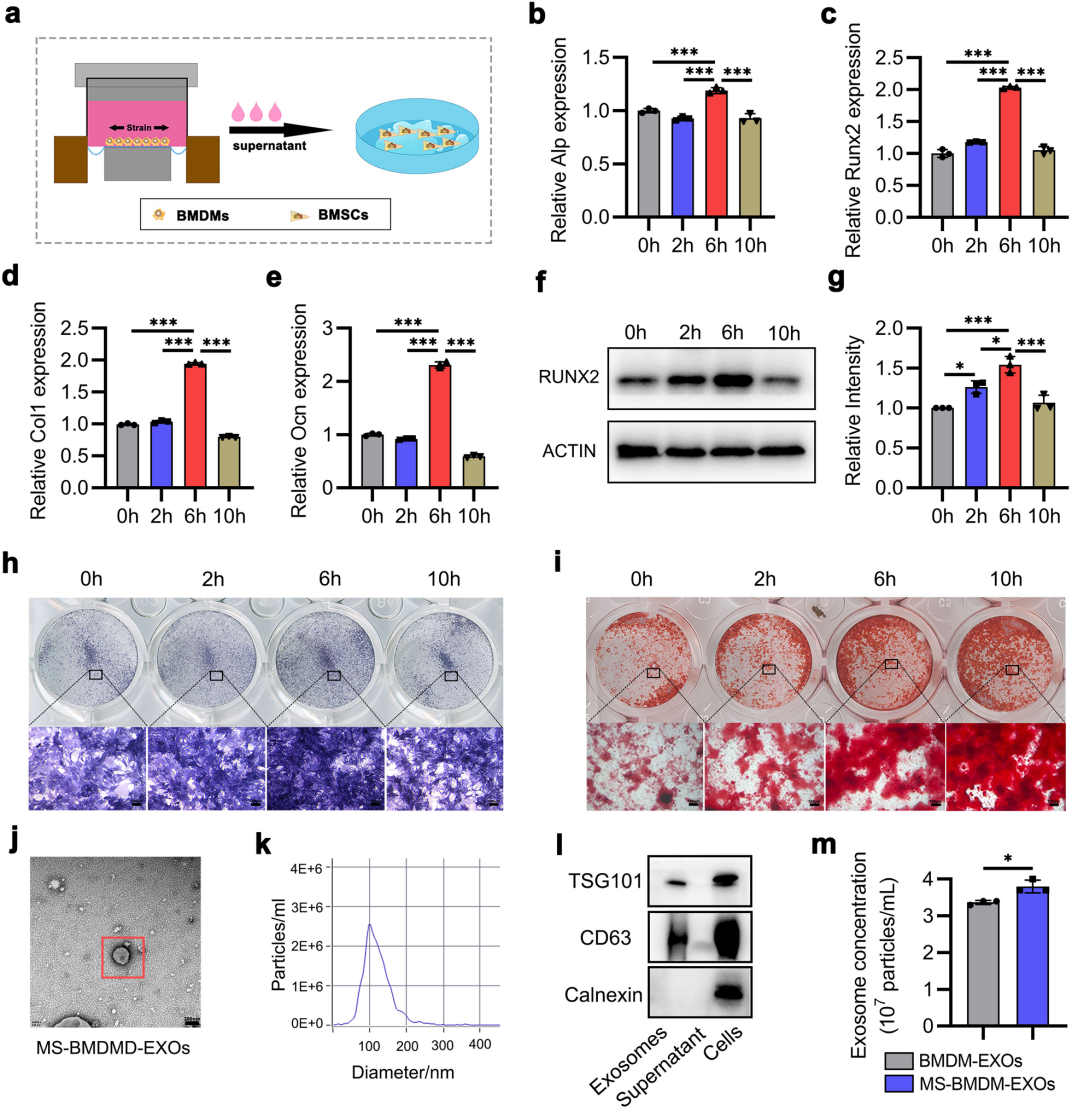
Mechanical force promotes BMDM-mediated BMSCs osteogenesis. **a** Schematic illustration. **b-f** qRT‒PCR analysis of the mRNA expression of osteogenic genes and Western blotting of RUNX2 protein levels in BMSCs after treatment with conditioned medium from MS-BMDMs (n = 3). **g** Relative intensity analysis from f (n = 3). **h, i** ALP staining and Alizarin red staining of BMSCs after treatment with conditioned medium from MS-BMDMs. Scale bar: 100 μm. **j** Representative TEM images of MS-BMDM-EXOs. Scale bar: 200 nm. **k** Particle size distribution of MS-BMDM-EXOs from NTA.** l** Western blotting of the exosomal markers CD63, and TSG101, and the cell marker Calnexin. **m** Concentration of BMDM-EXOs and MS-BMDM-EXOs from NTA (n = 3). Data are shown as the mean ± SD. Two-tailed unpaired Student’s t test or one-way ANOVA followed by Tukey’s post hoc multiple comparisons were performed. *P < 0.05, **P < 0.01, ***P < 0.001

Previous studies have shown that mechanical force could promote MSCs osteogenesis through the modulation of macrophages [25, 26]. To mimic BMSCs osteogenesis during OTM in vitro, we generated a mechanical force response culture system in which BMDMs were stimulated with different mechanical stimulations (MS), and their supernatants were then collected to treat BMSCs (Fig. 1a; Additional file 1: Fig. S1). We first measured the viability of BMDMs under the influences of different strain levels (0, 5, 10, and 15%) or durations (0, 2, 6, and 10 h) by CCK-8 analysis. After BMDMs were treated with different strain levels (0, 5, 10, and 15%) for 2 h, their cell viability was significantly increased under the 10% strain compared with other groups (Additional file 1: Fig. S2a). No significant differences were observed in BMDMs viability when they were treated with 10% mechanical force for 2, 6, and 10 h, respectively (Additional file 1: Fig. S2b). Therefore, 10% strain was used as the standard mechanical force for further experiments.

Next, we collected the supernatants from BMDMs pretreated with 10% strain for 0, 2, 6, and 10 h and prepared conditioned medium to culture BMSCs (Fig. 1a). qRT‒PCR showed that MS (10%, 6 h)-BMDMs-derived conditioned medium significantly increased osteogenic gene expression (Alp, Runx2 Col1 and Ocn) in BMSCs compared with that in other groups (Fig. 1b–e). Western blotting further confirmed that RUNX2, a central regulator of osteogenesis [27], was significantly upregulated in BMSCs after treatment with conditioned medium derived from MS (10%, 6 h)-BMDMs (Fig. 1f, g). Consistently, ALP staining also showed an increased ALP level in BMSCs treated with conditioned medium from MS (10%, 6 h)-BMDMs (Fig. 1h). Alizarin red staining revealed enhanced extracellular matrix mineralization deposition in the MS (10%, 10 h)-BMDM-treated BMSCs (Fig. 1i). In summary, these results confirmed that modest mechanical force promoted BMDM-mediated BMSC osteogenesis, and thus, MS (10%, 6 h) was used for the following experiment.

We set out to explore the player that may mediate BMSCs osteogenesis in the supernatants of MS-BMDMs. As a key intercellular mediator, exosomes derived from macrophages have been demonstrated to play an important role in modulating BMSCs osteogenesis [22, 28]. Therefore, we focused on the exosomes and collected the supernatants of the BMDMs to isolate exosomes after they were treated with MS (10%, 6 h). Transmission electron microscopy (TEM) revealed that these purified vesicles have cup- or sphere-shaped morphology (Fig. 1j), similar to the exosomes described previously [29]. Nanoparticle tracking analysis (NTA) demonstrated that the diameter of these particles predominantly ranged from 30 to 200 nm (Fig. 1k), which was consistent with the previously reported size distribution of exosomes [30]. Western blotting further verified that exosomal surface markers such as CD63 and TSG101 were present on these particles, while calnexin, a cytoplasmic protein, was not detected (Fig. 1l). NTA also showed that the supernatants of MS-BMDMs had a higher number of exosomes than BMDMs (Fig. 1m). All these data showed that these nanoparticles identified here were exosomes, and mechanical force promoted the secretion of exosomes derived from BMDMs. Based on these findings, we speculated that exosomes may be the key mediators secreted by MS-BMDMs to influence BMSCs osteogenesis.

### Exosomes derived from MS-BMDMs promote BMSCs osteogenesis in vitro

**Fig. 2:**
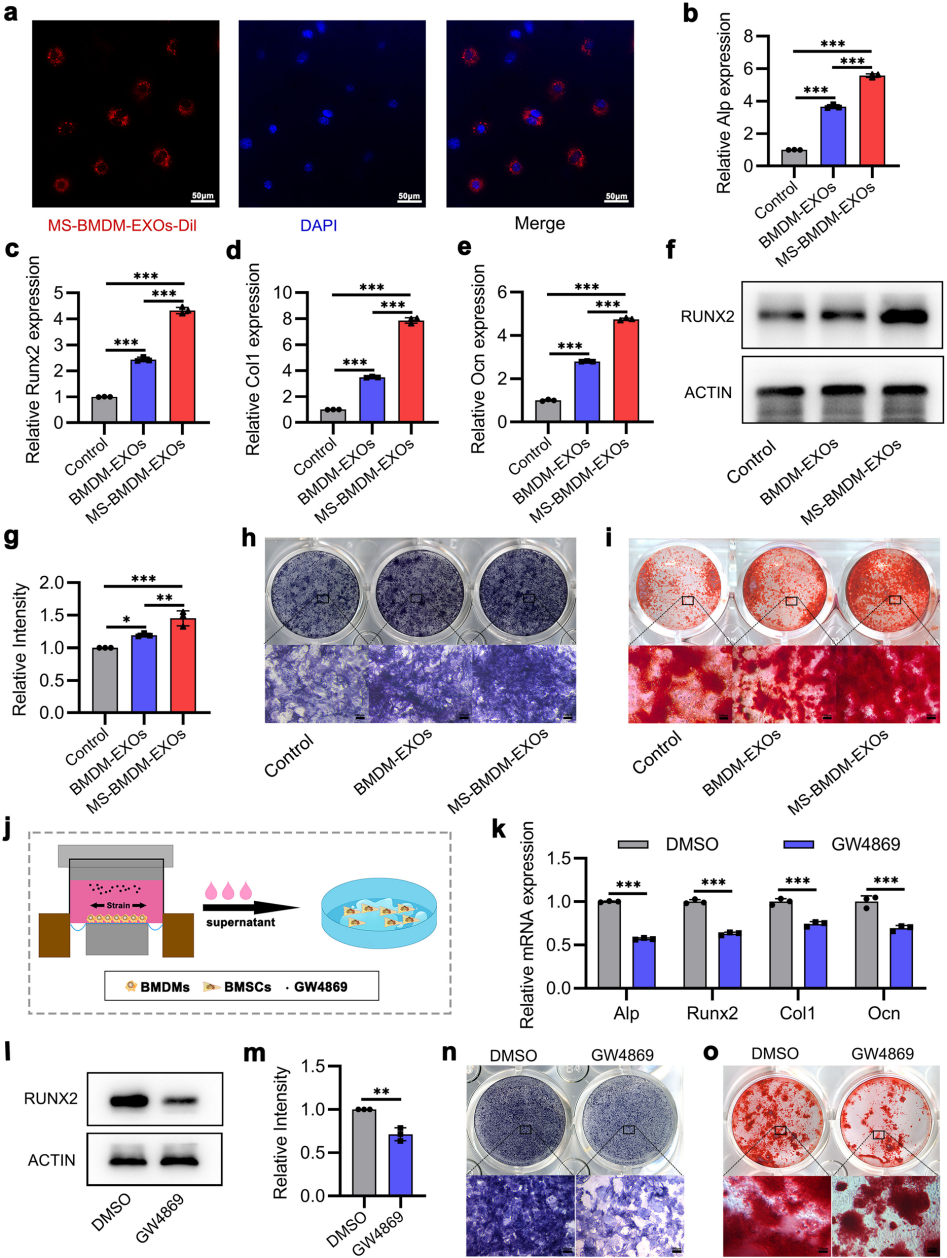
Exosomes derived from MS-BMDMs promote BMSCs osteogenesis in vitro. **a** Immunofluorescence images of the internalization of Dil-labelled MS-BMDM-EXOs in BMSCs after treatment for 12 h. MS-BMDM-EXOs and nuclei were stained with Dil (red) and DAPI (blue), respectively. Scale bar: 50 μm. **b-f** qRT‒PCR analysis of the mRNA expression of osteogenic genes and Western blotting of RUNX2 protein levels in BMSCs after treatment with PBS, BMDM-EXOs and MS-BMDM-EXOs (n = 3). **g** Relative intensity analysis of f (n = 3). **h, i** ALP staining and Alizarin red staining in BMSCs after treatment with PBS, BMDM-EXOs and MS-BMDM-EXOs. Scale bar: 100 µm. **j** Schematic illustration. **k, l** qRT‒PCR analysis of the mRNA expression of osteogenic genes and Western blotting of RUNX2 protein level in BMSCs after culture with conditioned medium from MS-BMDMs treated with GW4869 or DMSO as a control (n = 3). **m** Relative intensity analysis from l. **n, o** ALP staining and Alizarin red staining of BMSCs after culture with conditioned medium from MS-BMDMs treated with GW4869 or DMSO as a control. Scale bar: 100 μm. Data are shown as the mean ± SD. Two-tailed unpaired Student’s t test or one-way ANOVA followed by Tukey’s post hoc multiple comparisons were performed. *P < 0.05, **P < 0.01, ***P < 0.001

To confirm the potential effects of exosomes derived from MS-BMDMs in mediating BMSCs osteogenesis, we first labelled MS-BMDM-EXOs with the fluorescent dye Dil and treated BMSCs with these exosomes for 12 h. Fluorescence microscope imaging showed that these exosomes surrounded BMSCs nuclei, suggesting that they had been taken up by BMSCs (Fig. 2a). Next, BMSCs were treated with PBS, BMDM-EXOs or MS-BMDM-EXOs. qRT‒PCR and Western blotting showed that MS-BMDM-EXOs significantly promoted the mRNA expression of osteogenic genes (Alp, Runx2, Col1 and Ocn) and RUNX2 protein levels compared with PBS or BMDM-EXOs (Fig. 2b–g). ALP staining and Alizarin red staining also revealed that ALP level and extracellular matrix mineralization deposition were enhanced in BMSCs after treatment with MS-BMDM-EXOs compared with PBS or BMDM-EXOs (Fig. 2h, i). These results suggested that exosomes derived from MS-BMDMs promote osteogenesis in BMSCs.

Furthermore, GW4869, a widely used exosome secretion inhibitor [31, 32], was added to the culture medium of MS-BMDMs, and DMSO was used as a control (Fig. 2j). In BMSCs treated with conditioned medium from MS-BMDMs, GW4869 significantly decreased osteogenic gene (Alp, Runx2, Col1 and Ocn) and RUNX2 protein levels (Fig. 2k–m). Additionally, ALP staining and Alizarin red staining showed that ALP level and extracellular matrix mineralization deposition were also impaired when exosomes were blocked in MS-BMDMs (Fig. 2n, o). These results suggested that the key positive modulation of exosomes in MS-BMDMs regulated BMSCs osteogenesis.

### Exosomes derived from MS-BMDMs promote alveolar bone formation during OTM

**Fig. 3:**
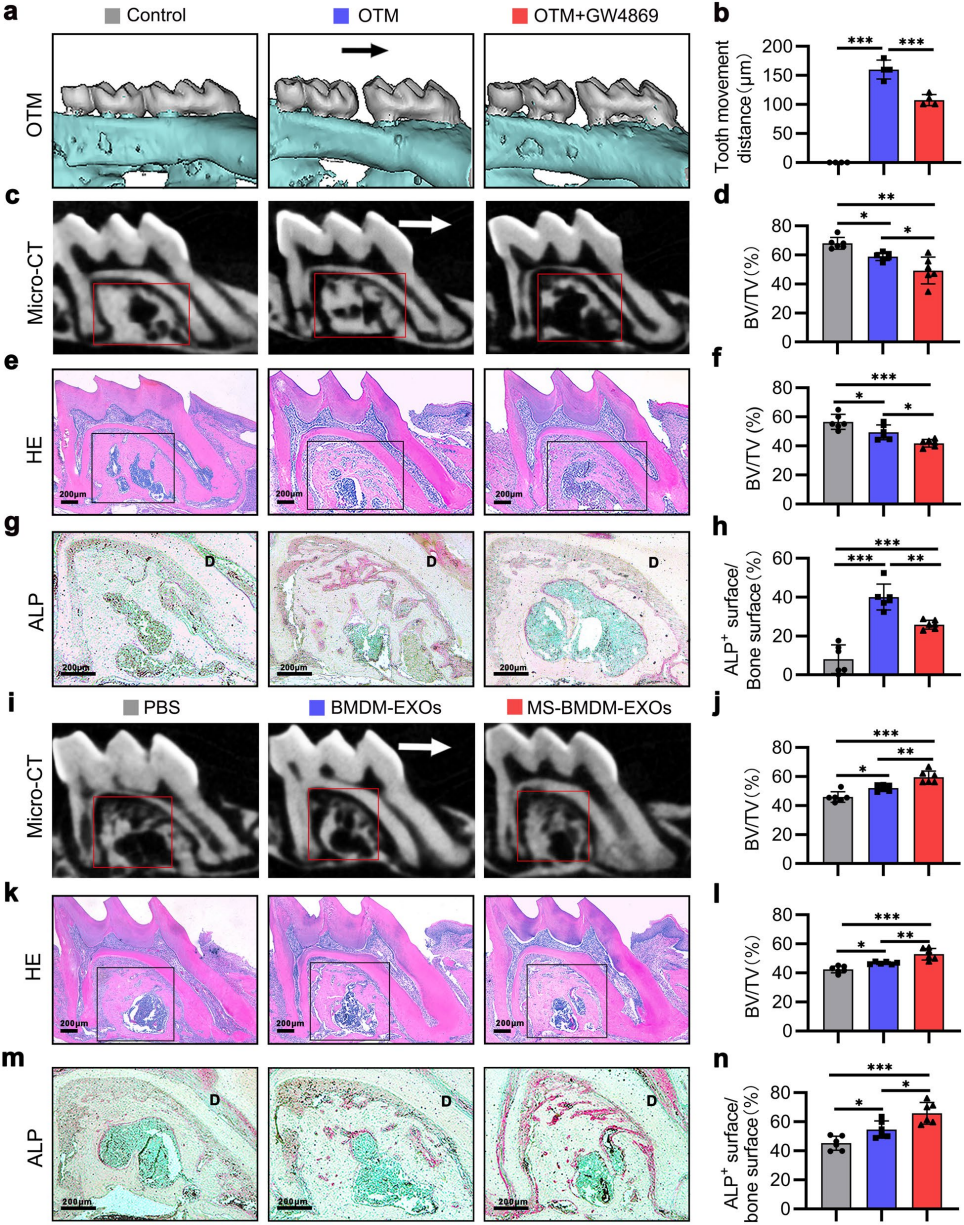
Exosomes derived from MS-BMDMs promote alveolar bone formation during OTM. **a** Representative 3D Micro-CT reconstruction images of the murine OTM model at day 14. Arrow: direction of tooth movement.** b** Quantification of tooth movement distance at day 14 after OTM treatment (n = 4). **c** Representative 3D scanned sections of the first molar at day 14 after OTM treatment. **d** Quantification from c. BV/TV of the interradicular region of the first molar (n = 6). **e** Representative HE staining images of the first molar at day 14 after OTM treatment. Scale bar: 200 μm. **f** Quantification from e. BV/TV of the interradicular region of the first molar (n = 6). **g** Representative ALP staining images of the interradicular region of the first molar at day 14 after OTM treatment. D: dentin. Scale bar: 200 μm. **h** Quantification from g. ALP-positive surface relative to bone surface (%) in the interradicular region of the first molar (n = 6).** i** Representative 3D scanned sections of the first molar at day 14 after OTM treatment. Arrow: direction of tooth movement. **j** Quantification from i. BV/TV of the interradicular region of the first molar (n = 6). **k** Representative HE staining images of the first molar at day 14 after OTM treatment. Scale bar: 200 μm. **l** Quantification from k. BV/TV of the interradicular region of the first molar (n = 6). **m** Representative ALP staining images of the interradicular region of the first molar at day 14 after OTM treatment. D: dentin. Scale bar: 200 μm. **n** Quantification from m. ALP-positive surface relative to bone surface (%) in the interradicular region of the first molar (n = 6). Data are shown as the mean ± SD. One-way ANOVA followed by Tukey’s post hoc multiple comparisons was performed. *P < 0.05, **P < 0.01, ***P < 0.001; ns, not significant

To explore the role of exosomes derived from MS-BMDMs in alveolar bone formation during OTM, we established an OTM model in 2-month-old mice as previously described along with intraperitoneal injection of GW4869 every 2 days [33] (Additional file 1: Fig. S3). After force was applied for 14 days, tooth movement was significantly suppressed by administration of GW4869 (Fig. 3a, b). Micro-CT analysis showed that the BV/TV of alveolar bone in the interradicular region of the loaded tooth was significantly decreased after GW4869 injection compared with the OTM group (Fig. 3c, d). These results were consistent with the HE staining results (Fig. 3e, f). In addition, ALP staining showed that the ALP-positive surface in the interradicular region and tension side of loaded alveolar bone was increased in the OTM group compared with the Control group, and it was suppressed after GW4869 injection (Fig. 3g, h; Additional file 1: Fig. S4), thereby indicating that exosome inhibition could impair alveolar bone formation during OTM. These findings suggest that exosomes are essential for mechanical force-induced alveolar bone formation during OTM.

To further evaluate the function of exosomes derived from MS-BMDMs in alveolar bone formation during OTM, PBS, BMDM-EXOs and MS-BMDM-EXOs were locally injected into the palatal gingiva of the loaded tooth every 3 days after the OTM model was established (Additional file 1: Fig. S5). After force was applied for 14 days, Micro-CT analysis revealed that BMDM-EXOs treatment greatly increased the BV/TV of alveolar bone in the interradicular region of the loaded tooth compared with PBS, and it was further enhanced by MS-BMDM-EXOs (Fig. 3i, j). These results were consistent with the HE staining results (Fig. 3k, l). In addition, through ALP staining, we found that the ALP-positive surface in the interradicular region and tension side of the loaded alveolar bone was also significantly increased after treatment with BMDM-EXOs compared with PBS, and this increase was further promoted by MS-BMDM-EXOs (Fig. 3m, n; Additional file 1: Fig. S6). These results suggested that exosomes derived from MS-BMDMs promoted alveolar bone formation during tooth movement.

### UCHL3 is enriched in MS-BMDM-derived exosomes

**Fig. 4:**
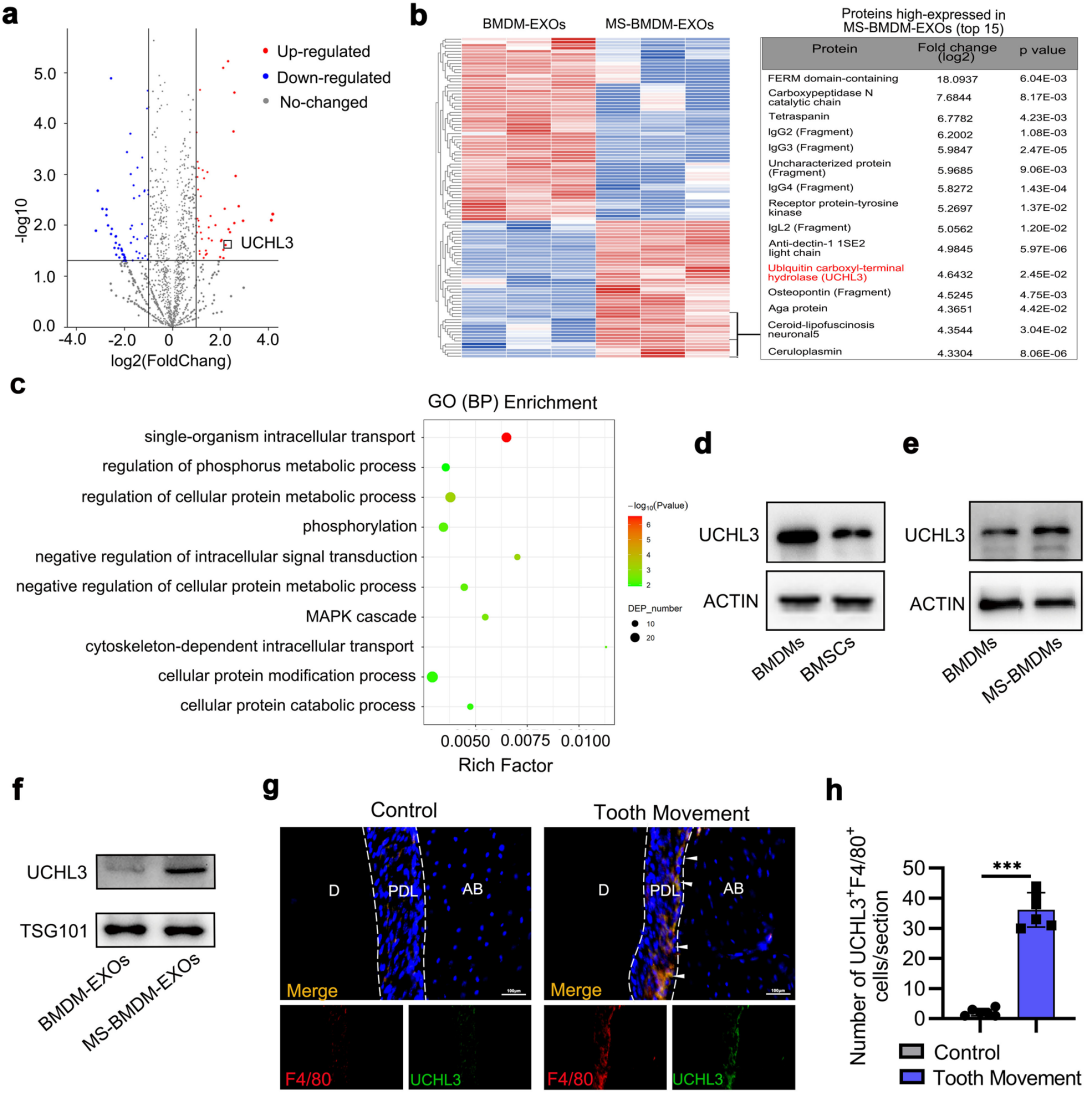
UCHL3 is enriched in MS-BMDM-derived exosomes. **a** Differentially expressed proteins between MS-BMDM-EXOs and BMDM-EXOs. Red, increased expression; blue, decreased expression; grey, no difference. *P* < 0.05 and fold change > 2 or < 0.5 were considered significant. **b** Heatmap diagram of differential protein expression between MS-BMDM-EXOs and BMDM-EXOs. Red, increased expression; blue, decreased expression; grey, no difference. Top 15 upregulated proteins in MS-BMDM-EXOs (UCHL3 was ranked eleventh). **c** GO Biological Process enrichment analysis of the differentially expressed proteins between MS-BMDM-EXOs and BMDM-EXOs. **d** Western blotting of UCHL3 protein levels in BMDMs and BMSCs. **e** Western blotting of UCHL3 protein levels in BMDMs and MS-BMDMs. **f** Western blotting of UCHL3 protein levels in BMDM-EXOs and MS-BMDM-EXOs. **g, h** Representative immunofluorescence staining and quantification of F4/80^+^ (red) UCHL3^+^ (green) cells in the control and OTM groups at 14 d after OTM treatment (n = 6). White arrows indicate F4/80^+^UCHL3^+^ cells. D: dentin, AB: Alveolar bone, PDL: periodontal ligament. Scale bar: 100 μm. Data are shown as the mean ± SD. Two-tailed unpaired Student’s t test was performed. ***P < 0.001; ns, not significant

We next analyzed the protein expression profiles in MS-BMDM-EXOs and BMDM-EXOs by proteomic analysis to explore the mechanism by which MS-BMDM-EXOs mediate BMSC osteogenesis. Comparing the protein expression levels in MS-BMDM-EXOs and BMDM-EXOs with a fold change > 2 or < 0.5 and *P* < 0.05 as the threshold cut-off, we identified 45 upregulated and 60 downregulated proteins, respectively, in the MS-BMDM-EXOs group (Fig. 4a, b). Subsequently, differentially expressed proteins were selected for bioinformatic analysis. Gene ontology (GO) enrichment analysis revealed that these proteins were involved in multiple biological processes in which cell protein modification and MAPK cascades were related to osteogenesis (Fig. 4c). Among all the 45 upregulated proteins, we identified UCHL3, which is ranked 11^th^ among the upregulated proteins and participates in the cellular protein modification process (Fig. 4b). Previous studies demonstrated that UCHL3, a deubiquitinase, is a critical regulator of osteogenesis [34]. Our Western blotting results further revealed that the UCHL3 protein level was higher in BMDMs than in BMSCs, mechanical force increased the protein level of UCHL3 in BMDMs and BMDM-EXOs (Fig. 4d–f). And after treatment with BMDM-EXOs and MS-BMDM-EXOs, the UCHL3 protein level in BMSCs was improved (Additional file 1: Fig. S7). In addition, immunofluorescence staining showed that the number of UCHL3^+^F4/80^+^ macrophages was significantly increased after force application (Fig. 4g, h). Therefore, UCHL3 may be one of the most important factors in MS-BMDM-EXOs, and we selected it for further analyses (Additional file 1: Fig. S8).

### UCHL3 is required for MS-BMDM-EXO-mediated BMSCs osteogenesis in vitro

**Fig. 5:**
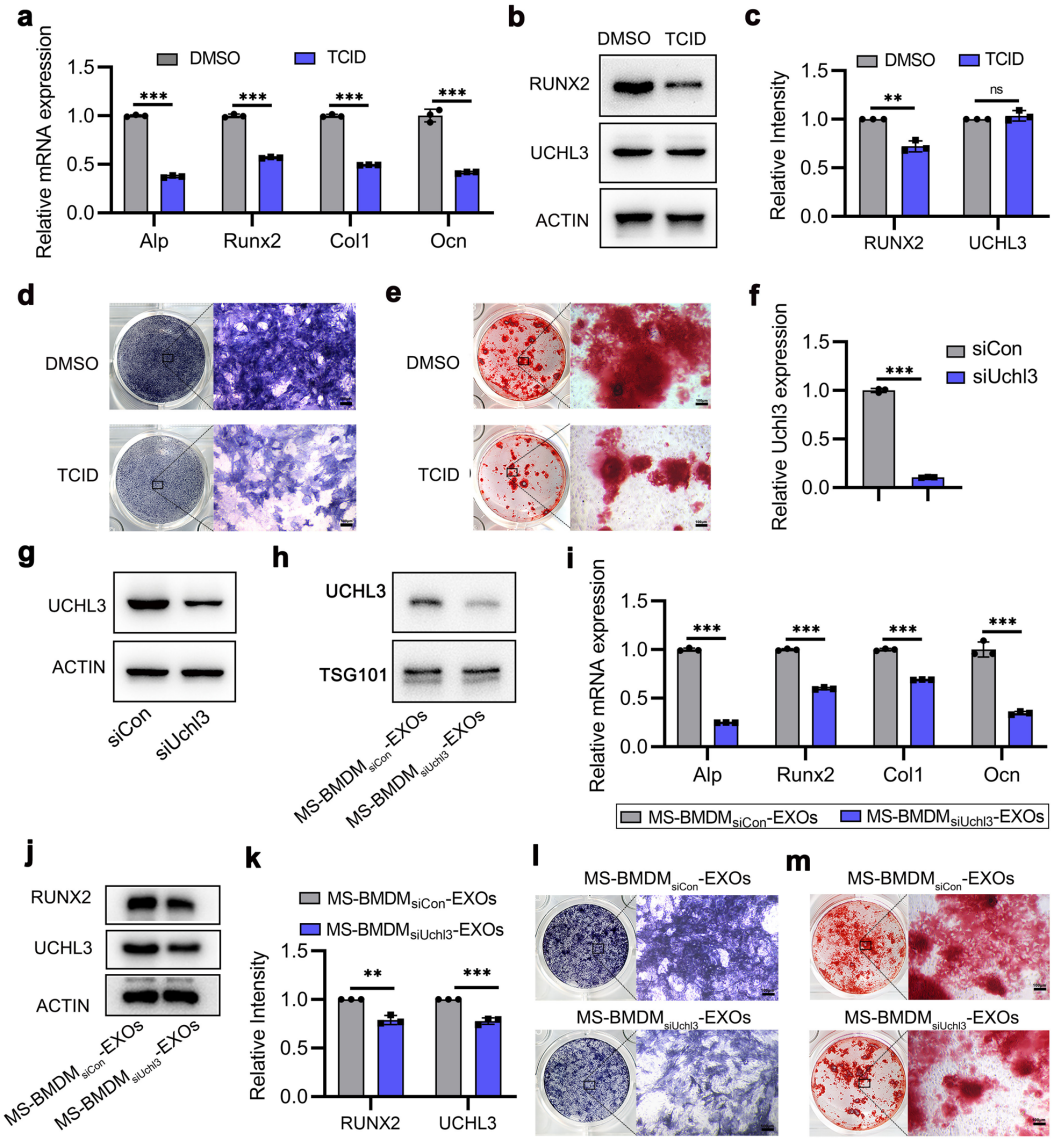
UCHL3 is required for MS-BMDM-EXO-mediated BMSC osteogenesis in vitro. **a, b** qRT‒PCR of the mRNA expression of osteogenesis genes and Western blotting of RUNX2 and UCHL3 protein levels in BMSCs after treatment with UCHL3 inhibitor TCID, and DMSO as a control (n = 3). **c** Relative intensity analysis of b (n = 3). **d, e** ALP staining and Alizarin red staining in BMSCs after treatment with TCID, and DMSO as a control. Scale bar: 100 μm. **f, g** qRT‒PCR of the mRNA expression of Uchl3 and Western blotting of UCHL3 protein level in MS-BMDMs after treatment with siCon and siUchl3 (n = 3). **h** Western blotting of UCHL3 protein level in exosomes derived from MS-BMDMs after treatment with siCon and siUchl3. **i, j** qRT‒PCR of the mRNA expression of osteogenic genes and Western blotting of UCHL3 and RUNX2 protein levels in BMSCs after treatment with MS-BMDMs_siCon_-EXOs and MS-BMDMs_siUchl3_-EXOs. **k** Relative intensity analysis of j (n = 3). **l, m** ALP staining and Alizarin red staining in BMSCs after treatment with MS-BMDMs_sicon_-EXOs and MS-BMDMs_siUchl3_-EXOs. Two-tailed unpaired Student’s t test was performed. **P < 0.01, ***P < 0.001; ns, not significant

To examine the role of UCHL3 in MS-BMDM-EXO-mediated BMSC osteogenesis, we first used TCID, a small molecule inhibitor which could inhibit the deubiquitinase activity of UCHL3 [35], to treat BMSCs. qRT‒PCR and Western blotting showed that TCID treatment significantly reduced osteogenic gene (Alp, Runx2 Col1 and Ocn) and RUNX2 protein levels (Fig. 5a–c). ALP staining and Alizarin red staining revealed that TCID also diminished ALP level and extracellular matrix mineralization deposition in BMSCs (Fig. 5d, e). These results suggested that UCHL3 was critical for the osteogenesis of BMSCs.

To further confirm the function of UCHL3 in MS-BMDM-EXO-mediated BMSC osteogenesis, we used siUchl3 to knockdown Uchl3 expression in MS-BMDMs. qRT‒PCR and Western blotting showed that it had an effective inhibitory efficiency on the mRNA and protein levels of UCHL3 (Fig. 5f, g). After MS-BMDMs were transfected with siUchl3, their supernatants were collected to isolate exosomes. Western blotting also revealed an effective inhibitory efficiency of siUchl3 for UCHL3 protein levels in MS-BMDM_siUchl3_-EXOs (Fig. 5h). BMSCs were then treated with MS-BMDM_siCon_-EXOs and MS-BMDM_siUchl3_-EXOs. qRT‒PCR and Western blotting showed that after UCHL3 was downregulated in MS-BMDM-EXOs, osteogenic gene expression (Alp, Runx2, Col1 and Ocn) and the protein level of RUNX2 in BMSCs were also significantly downregulated compared with those in the MS-BMDM_siCon_-EXOs group (Fig. 5i–k). Additionally, through ALP staining and Alizarin red staining, it was found that ALP level and extracellular matrix mineralization deposition were also impaired when UCHL3 was inhibited in MS-BMDM-EXOs (Fig. 5l, m). These results indicated that UCHL3 was required for MS-BMDM-EXO-mediated BMSC osteogenesis.

### UCHL3 is required for MS-BMDM-EXO-mediated alveolar bone formation during OTM

**Fig. 6:**
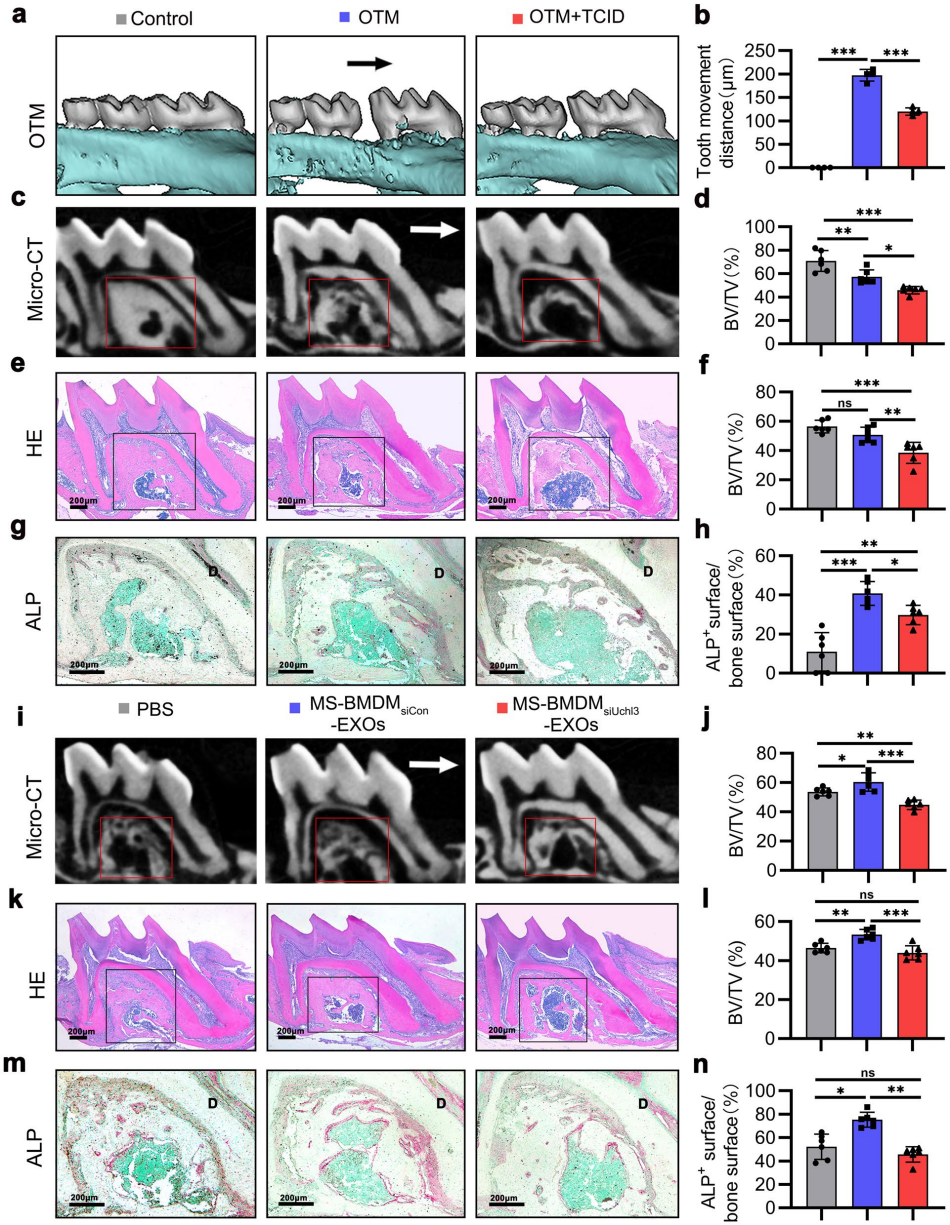
UCHL3 is required for MS-BMDM-EXO-mediated alveolar bone formation during OTM. **a** Representative 3D Micro-CT reconstruction images of the murine OTM model at day 14 after OTM treatment. Arrow: direction of tooth movement. **b** Quantification of tooth movement distance at day 14 after OTM treatment (n = 4). **c** Representative 3D scanned sections of the first molar at day 14 after OTM treatment. **d** Quantification from c. BV/TV of the interradicular region of the first molar (n = 6). **e** Representative HE staining images of the first molar at day 14 after OTM treatment. Scale bar: 200 μm. **f** Quantification from e. BV/TV of the interradicular region of the first molar (n = 6). **g** Representative ALP staining images of the interradicular region of the first molar at day 14 after OTM treatment. D: dentin. Scale bar: 200 μm. **h** Quantification from g. ALP-positive surface relative to bone surface (%) in the interradicular region of the first molar (n = 6). **i** Representative 3D scanned sections of the first molar at day 14 after OTM treatment. Arrow: direction of tooth movement. **j** Quantification from i. BV/TV of the interradicular region of the first molar (n = 6). **k** Representative HE staining images of the first molar at day 14 after OTM treatment. Scale bar: 200 μm.** l** Quantification from k. BV/TV of the interradicular region of the first molar (n = 6). **m** Representative ALP staining images of the interradicular region of the first molar at day 14 after OTM treatment. D: dentin. Scale bar: 200 μm.** n** Quantification from m. ALP-positive surface relative to bone surface (%) in the interradicular region of the first molar. Data are shown as the mean ± SD. One-way ANOVA followed by Tukey’s post hoc multiple comparisons was performed. *P < 0.05, **P < 0.01, ***P < 0.001; ns, not significant

To identify the unique role of UCHL3 derived from MS-BMDM-EXOs in mediating alveolar bone formation during OTM, we first established an OTM model and performed intraperitoneal injection of the UCHL3 inhibitor TCID every 2 days during loading (Additional file 1: Fig. S9). After 14 days of force application, the tooth movement distance significantly decreased after TCID injection (Fig. 6a, b). Micro-CT analysis showed that compared with the OTM group, the BV/TV of alveolar bone in the interradicular region of the loaded tooth decreased after TCID injection (Fig. 6c, d). This phenomenon was consistent with the HE staining results (Fig. 6e, f). In addition, ALP staining showed that the ALP-positive surface in the interradicular region and tension side of loaded alveolar bone were significantly increased in the OTM group compared with the Control group, and they were suppressed after TCID injection (Fig. 6g, h; Additional file 1: Fig. S10). These findings indicated that UCHL3 was required for mechanical force-induced bone formation during OTM.

To further explore the function of UCHL3 in MS-BMDM-EXO-mediated alveolar bone formation during OTM, PBS, MS-BMDM_siCon_-EXOs, and MS-BMDM_siUchl3_-EXOs were locally injected into the palatal gingiva of the loaded tooth every 3 days after the OTM model was established (Additional file 1: Fig. S11). After 14 days, Micro-CT analysis revealed that after UCHL3 was downregulated in MS-BMDM-EXOs, the BV/TV of alveolar bone in the interradicular region of the loaded tooth was also reduced compared with MS-BMDM_siCon_-EXOs (Fig. 6i, j). These results were consistent with the HE staining results (Fig. 6k, l). In addition, ALP staining showed that the ALP-positive surface in the interradicular region and tension side of loaded alveolar bone was also significantly impaired when UCHL3 was inhibited in MS-BMDM-EXOs (Fig. 6m, n; Additional file 1: Fig. S12). Collectively, these results indicated that UCHL3 was required for MS-BMDM-EXO-mediated alveolar bone formation during OTM.

### MS-BMDM‑derived exosomal UCHL3 promotes BMSCs osteogenesis by targeting SMAD1

**Fig. 7:**
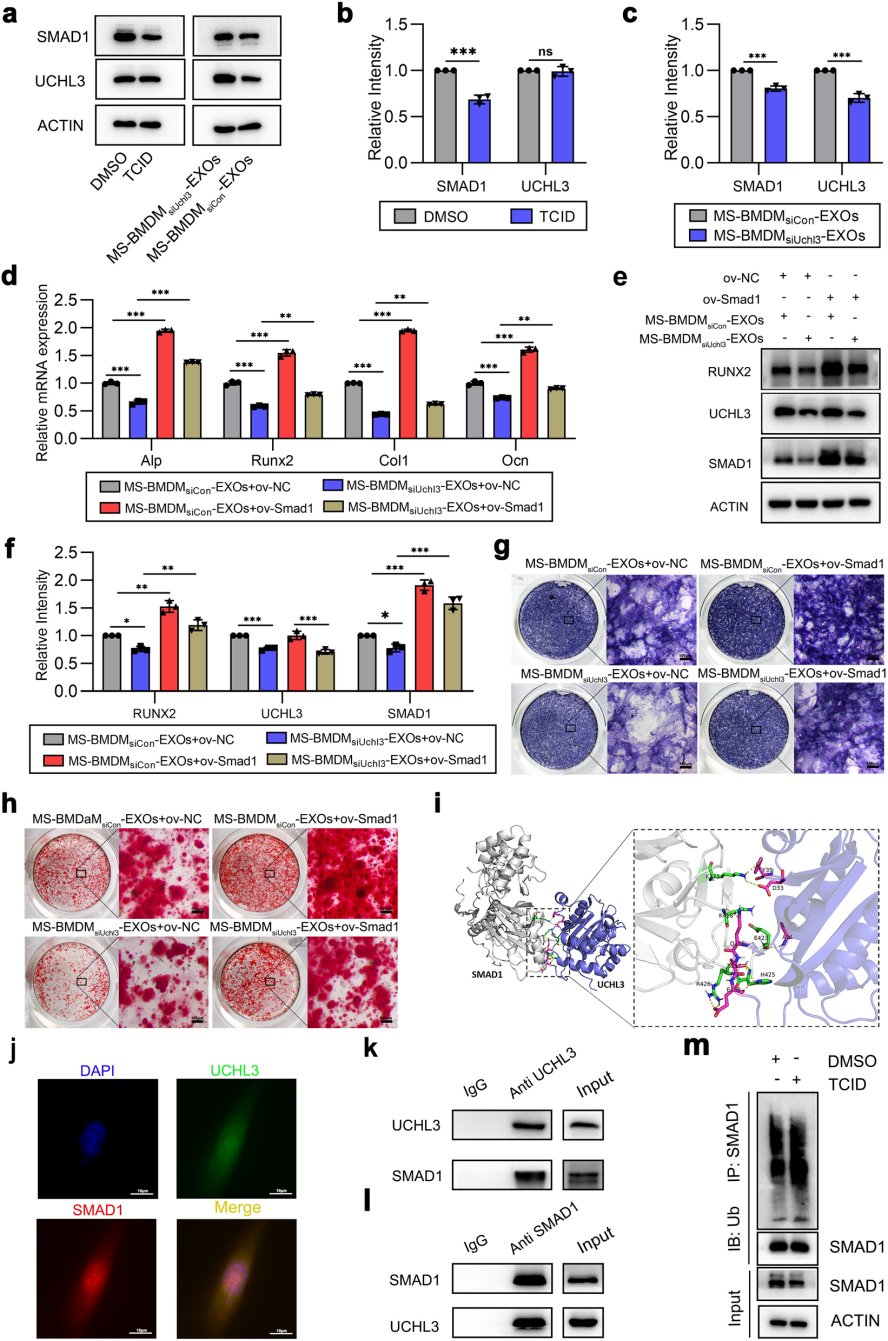
MS-BMDM‑derived exosomal UCHL3 promotes BMSCs osteogenesis by targeting SMAD1. **a** Western blotting of SMAD1 and UCHL3 protein levels in BMSCs after treatment with the UCHL3 inhibitor TCID or MS-BMDM_siUchl3_-EXOs. **b, c** Relative intensity analysis of a (n = 3). **d, e** qRT‒PCR of the mRNA expression of osteogenesis genes and Western blotting of RUNX2, UCHL3 and SMAD1 protein levels in BMSCs after overexpression of Smad1 with lentiviruses and treatment with MS-BMDM_siUchl3_-EXOs (n = 3). **f** Relative intensity analysis of e (n = 3). **g, h** ALP staining and Alizarin red staining in BMSCs after overexpression of Smad1 with lentiviruses and treatment with MS-BMDM_siUchl3_-EXOs. Scale bar: 100 μm. **i** 3D modelling of the interaction between UCHL3 (blue) and SMAD1 (grey) proteins predicted by protein‒protein docking from Alphafold 2. The stick structure represents amino acid residues, and the yellow dashed lines represent hydrogen bonds. **j** Immunofluorescence images of the colocalization of SMAD1 and UCHL3 in BMSCs (green UCHL3, red SMAD1, blue DAPI). Scale bar: 10 μm. **k** Endogenous UCHL3 and SMAD1 proteins interacted in BMSCs. UCHL3 protein was immunoprecipitated with anti-UCHL3 antibody. IgG served as a negative control, and endogenous SMAD1 was measured by Western blotting. **l** Endogenous UCHL3 and SMAD1 proteins interacted in BMSCs. SMAD1 protein was immunoprecipitated with anti-SMAD1 antibody. IgG served as a negative control, and endogenous UCHL3 was measured by Western blotting. **m** Western blotting of the polyubiquitinated level of SMAD1 in BMSCs after treatment with TCID. SMAD1 protein was immunoprecipitated, and polyubiquitinated SMAD1 protein was measured by Western blotting using anti-Ub antibody. Data are shown as the mean ± SD. Two-tailed unpaired Student’s t test or one-way ANOVA followed by Tukey’s post hoc multiple comparisons were performed. *P < 0.05, **P < 0.01, ***P < 0.001; ns, not significant

We next explored the mechanism by which MS-BMDM-derived exosomal UCHL3 modulates BMSCs osteogenesis. It has been reported that UCHL3 can interact with SMAD1, a classical osteogenesis-related molecule involved in BMSC osteogenesis [34, 36, 37]. Thus, we hypothesized that MS-BMDM-derived exosomal UCHL3 could mediate BMSC osteogenesis through the regulation of SMAD1. To verify the correlation between UCHL3 and SMAD1, we used TCID and MS-BMDM_siUchl3_-EXOs to treat BMSCs and found that the protein level of SMAD1 was downregulated when UCHL3 was knocked down (Fig. 7a–c). Next, BMSCs overexpressing SMAD1 with lentiviruses were treated with MS-BMDM_siCon_-EXOs and MS-BMDM_siUchl3_-EXOs. qRT‒PCR and Western blotting revealed an effective overexpression efficiency of SMAD1 in BMSCs (Additional file 1: Fig. S13). Overexpression of SMAD1 significantly reversed the downregulated osteogenic gene expression (Alp, Runx2, Col1, and Ocn) and protein level of RUNX2 caused by UCHL3 inhibition in MS-BMDM-EXOs (Fig. 7d–f). ALP staining and Alizarin red staining further confirmed a similar rescue effect of SMAD1 overexpression in promoting the decreased ALP level and extracellular matrix mineralization deposition caused by UCHL3 inhibition in MS-BMDM-EXOs (Fig. 7g, h).

Furthermore, we wondered how UCHL3 modulated the function of SMAD1 in BMSCs. The protein‒protein docking results from Alphafold 2 revealed that UCHL3 could directly bind with SMAD1 through hydrogen bonds (Fig. 7i). Since UCHL3 is a regulator of ubiquitin, we considered that UCHL3 may increase SMAD1 levels by regulating its degradation. Further immunofluorescence and co-immunoprecipitation (Co-IP) verified the interaction between UCHL3 and SMAD1 in BMSCs (Fig. 7j–l). In addition, inhibition of endogenous UCHL3 by TCID elevated the ubiquitination level of endogenous SMAD1 in BMSCs (Fig. 7m), which indicated that UCHL3 could elevate SMAD1 protein stabilization through deubiquitination. Meanwhile, the immunohistochemical staining experiment showed that UCHL3 inhibitor TCID reduced the SMAD1 level in the loaded alveolar bone in the tooth movement mice (Additional file 1: Fig. S14). In summary, these results indicated that SMAD1 was necessary for MS-BMDM-EXO-derived UCHL3 to promote BMSCs osteogenesis.

## Discussion

Orthodontic treatment is an effective way to correct malocclusion. The essence of orthodontic treatment is a local aseptic inflammation-associated alveolar bone remodelling process induced by mechanical force [5]. Previous studies have shown that during tooth movement, macrophages secrete inflammatory cytokine and modulate the alveolar bone remodeling inflammatory microenvironment [14, 15]. Nevertheless, the mechanism by which macrophages regulate mechanical force-induced alveolar bone remodelling during OTM is not clear. In the present study, we demonstrated that the exosomes derived from mechanical force-stimulated macrophages promote BMSCs osteogenesis, thereby promoting alveolar bone formation during OTM. Furthermore, we verified that exosomal UCHL3, by targeting SMAD1, plays a prominent role in MS-BMDM-EXO-mediated pro-osteogenesis. In summary, this study demonstrated the mechanism of mechanical force-induced alveolar bone formation during OTM from the perspective of macrophages regulating BMSCs osteogenesis through exosomes.

Macrophages can regulate BMSCs osteogenesis by secreting proinflammatory factors, e.g., TNF-α, or anti-inflammatory factors, e.g., IL-10 [38]. Wei et al. found that under mechanical stimulation, macrophages polarized to the M2 phenotype and produced anti‐inflammatory cytokines such as IL‐10 and TGF‐β to regulate the local inflammatory microenvironment and promote BMSC osteogenesis [25, 26]. Our study confirmed the promotive effect of force stimulation on BMDM-mediated BMSC osteogenesis. Exosomes, which contain miRNA, protein and nucleic acids, are important mediators between macrophages and BMSCs [39]. Cumulative studies have recently reported that under the stimulation of some biomimetic materials, exosomes derived from macrophages promote BMSCs osteogenesis [32, 40]. However, whether mechanical force regulates BMSCs osteogenesis via macrophage-derived exosomes remains unclear. Our study showed that BMSCs osteogenesis was reduced when exosomes derived from MS-BMDMs were inhibited. Compared with exosomes derived from BMDMs, exosomes derived from MS-BMDMs showed a stronger ability to promote BMSCs osteogenesis. Therefore, it is reasonable to conclude that mechanical force could regulate macrophage-derived exosomes to promote BMSCs osteogenesis.

Orthodontic tooth movement follows Wolff's law—the remodelling of bone and surrounding tissues is an adaptive biological response to the external force [41]. Xu et al. recently found that in mechanical force-induced alveolar bone remodelling, macrophages could respond to force stimulation through Piezo1 [42]. However, knowledge of how force-mediated macrophages regulate alveolar bone remodeling is still insufficient. Evidence suggests that exosomes derived from macrophages are involved in bone formation in fracture models [22], periodontitis models [43], and osteoporosis models [44]. In this study, using the exosome inhibitor GW4869 and exosomes derived from macrophages to treat tooth movement mouse models, we demonstrated that mechanical force-regulated macrophage-derived exosomes could promote alveolar bone formation during OTM.

Accumulating evidence has proved that exosomes are mechanosensitive [45]. Our study confirmed that exosomes are indeed mechanosensitive and discovered that there was a higher concentration and different cargo of macrophage-derived exosomes under mechanical stimulation. To explore the mechanism by which MS-BMDM-EXOs mediate BMSCs osteogenesis, we performed a proteomic study and found that MS-BMDM-EXOs contain much higher levels of the deubiquitinase UCHL3. Deubiquitinases, which oppose protein ubiquitination by hydrolyzing ubiquitin linkages, control the function or abundance of targeted proteins and influence physiological or pathological processes [46, 47]. While UCHL3 has been identified as a novel mediator in DNA repair [48], aerobic glycolysis [49], and cancer progression [50], its role in bone formation has not been clarified. Kim et al. verified the positive effect of UCHL3 on BMSCs osteogenesis [34]. However, whether UCHL3 derived from MS-BMDM-EXOs plays a role in BMSCs osteogenesis and mechanical force-induced alveolar bone formation remains unknown. We used the UCHL3 inhibitor TCID and UCHL3^low^ exosomes from MS-BMDMs to treat BMSCs and tooth movement mouse models and observed that BMSCs osteogenesis and alveolar bone formation were reduced by TCID and UCHL3^low^ exosomes.

SMAD1, an immediate downstream molecule of BMP receptors, is involved in the regulation of BMSCs osteogenesis by affecting BMP signal transduction [51–53]. It has been reported that UCHL3, which decreases the amount of polyubiquitinated SMAD1, might be critical for osteogenesis [34]. Consistently, our data showed that UCHL3 physically interacted with SMAD1 and that its expression in BMSCs was positively correlated with SMAD1. Overexpression of SMAD1 rescued the effect of inhibiting MS-BMDM-derived exosomal UCHL3 on BMSCs osteogenesis. Moreover, using a ubiquitination assay, we confirmed that UCHL3 decreases the amount of polyubiquitinated SMAD1. This finding indicated that UCHL3 promotes BMSCs osteogenesis by decreasing SMAD1 ubiquitination degradation.

Notably, we only characterized the protein profile in MS-BMDM-EXOs. It is possible that mRNAs, miRNAs or other noncoding RNAs in exosomes also play a key role in this pro-osteogenesis process. Moreover, although we found that MS-BMDM-derived exosomal UCHL3 promotes BMSCs osteogenesis by targeting SMAD1, additional questions, such as binding sites, still require clarification. Therefore, further studies are needed to evaluate the mechanism by which UCHL3 regulates SMAD1.

## Conclusions

In summary, we showed that mechanical force could alter the protein composition in macrophage-derived exosomes. Transfer of UCHL3 by exosomes from mechanically stimulated macrophages to BMSCs improved SMAD1 levels in BMSCs, which was beneficial for BMSCs osteogenesis, thereby promoting alveolar bone formation during OTM. These findings therefore demonstrated the mechanism of mechanical force-induced osteogenesis during OTM from the perspective of macrophage-derived exosomes regulating BMSCs osteogenesis, which may contribute to the development of new solutions to clinical issues in orthodontic treatment.

## Methods

### Animals

C57BL/6 J mice (male, 8 weeks old) that used in the present study were from the Laboratory Animal Center of Nanjing Medical University. All experimental protocols were approved by the Committee of Nanjing Medical University for Animal Resources (approval ID: IACUC1601118).

### Application of orthodontic devices

Appropriate mechanical force was exerted on the molars of these mice for 14 days as described previously [33]. Briefly, the left maxillary first molar was ligated to the maxillary incisors by utilizing a nickel-titanium coil spring. The springs were activated to deliver the orthodontic force of 10 g. Every 24 h, the devices were reviewed and, if necessary, reinstalled. To alleviate pain, the mice were offered soft food. After 14 days, the mice were sacrificed for following experiments.

### Drugs and exosomes administration

For exosomes inhibitor treatment, mice were injected intraperitoneally with GW4869 (#HY-19363, MCE, USA) at one dose of 2.5 mg/kg every 2 days following the orthodontic device application. The same volume of DMSO was injected as that for the controls [54, 55].

For UCHL3 inhibitor treatment, mice were injected intraperitoneally with TCID (#HY-18638, MCE, USA) at one dose of 10 mg/kg every 2 days following the orthodontic device application. The same volume of DMSO was injected as that for the controls.

For exosomes treatment, injection of 10 μl (2 μg/μl) exosomes were given locally into the palatal gingiva of the loaded first maxillary molar every 3 days utilizing a 33-gauge needle Hamilton syringe (Hamilton Company, USA) [43, 56].

### Micro‑CT analysis

After being separated and fixed for 2 days in 4% paraformaldehyde, maxillae of mice were scanned by a Micro-CT scanner (Skyscan 1176, Bruker) at a resolution of 10 μm. The Micro-CT data were input into DataViewer (Bruker) to carry out 3D reconstruction, and meanwhile they were imported to CTAn (Bruker) to perform quantitative analysis. After that, the region of interest was selected from the inter-radicular region of the maxillary first molar and bone structures parameter BV/TV was analyzed [6, 57]. Then the Micro-CT data were imported into mimics (V20.0, Materialise, Belgium) for reconstruction and tooth movement distance measurement. Using the occlusal view of the 3D-reconstructed image, we obtained the midpoints of the distal marginal ridge of the first molar and the mesial marginal ridge of the second molar. The gap between the two points was the tooth movement distance [58].

### Histologic and immunofluorescent staining

After being isolated, maxilla was fixed for 2 days in 4% paraformaldehyde and decalcified for 28 days in 10% EDTA solution. Then, the samples were embedded in paraffin, where they were sectioned into 4 μm sections. Subsequently, the paraffin sections were stained using H&E for routine histology and bone structures parameter BV/TV analysis. ALP staining was also performed to identify osteoblasts as previously described [59].

For tissue immunofluorescent staining, the samples were embedded in Tissue-Tek O.C.T and sectioned into 8 μm sections. After being washed with PBS for 30 min and blocked with 10% BSA for 30 min at room temperature, the sections were incubated with primary antibodies against UCHL3 (1:200, #D25E6, Cell Signaling Technology, USA) overnight at 4 °C. Then the sections were washed with PBS, and incubated with fluorescein Cy3-conjugated secondary antibody (Beyotime, China) and fluorescein CoraLite488-conjugated secondary antibody (#SA00013-2, Proteintech, USA) for 1.5 h. Nuclei were counterstained with 4′,6-diamidino-2-phenylindole (DAPI, # H-1200, Vectorlabs, USA). Images were captured with a fluorescent microscope (Carl Zeiss).

There were two regions of interest: (1) the distal side of the mesial root of the maxillary first molar was used to characterize bone formation on the tension-side bone; (2) the inter-radicular region of the maxillary first molar was used to characterize general bone formation [6]. Quantification of the ALP-positive surface relative to the bone surface (%) was determined by ImageJ software (V1.8.0, National Institutes of Health, USA) [60].

### Cell culture

The bone marrow‐derived macrophages were isolated and cultured as described previously [60]. Specifically, the bone marrow cells isolated from the femurs and tibias of 4- to 6-week-old mice were cultured in the α-MEM medium added with 10% fetal bovine serum (ScienCell, USA), 1% penicillin/streptomycin (Gibco), and 25 ng/ml M-CSF (Peprotech, USA) for 5 days.

The bone marrow-derived mesenchymal stem cells (BMSCs) were isolated and cultured as described previously [61]. In brief, the bone marrow cells were first flushed out from the femurs and tibias of 4-week-old mice using α-MEM medium containing 12% FBS (ScienCell, USA) and 1%penicillin/streptomycin (Gibco, USA). After that, the flushing fluid was moved into a 6 cm-culture dish and cultured with BMSCs normal culture medium above. BMSCs in the second or the third passages were used in the subsequent experiments.

### Mechanical stretch of BMDMs and BMDMs transfection

For the mechanical stretch studies, BMDMs were cultured in flexible-bottom six-well culture plates (Flexcell International Corporation, USA) with a density of 1 × 10^6^ cells for each well. At the fifth day, BMDMs were treated with cyclic stretching at 0.5 Hz with different strains for specific durations using the FX-5000 T Flexcell Tension System (Flexcell International Corporation, USA). After mechanical stimulation, culture medium of BMDMs was refreshed. Two days later, the supernatants were collected for the following experiments.

For BMDMs transfection, we transfected BMDMs with 100 nM siRNA using Lipofectamine 2000 (#11668-019, Invitrogen, USA) following the instruction of the manufacturer after they were mechanically stimulated. Then 48 h later, the medium was replaced with medium containing EXOs-free FBS. And after another 48 h, supernatants were collected for isolating exosomes. The sequences were as follows: Uchl3-siRNA, 5′-GAACAGAAGAGGAAGAAAATT-3′ and 5′-UUUUCUUCCUCUUCUGUUCTT-3′, negative control Con-siRNA, 5′-UUCUCCGAACGUGUCACGUTT-3′ and 5′-ACGUGACACGUUCGGAGAATT-3′.

### Conditioned medium and exosomes treatment of BMSCs

After mechanical stimulation, culture medium of BMDMs was refreshed with medium containing EXOs-free FBS. Two days later, the supernatants were collected and conditioned medium was prepared by mixing the collecting BMDMs supernatants with normal BMSCs culture/osteogenic medium (#MUXMX-90021, Cyagen Biosciences, USA) at a ratio of 1:3. BMSCs were cultured with conditioned medium or medium containing exosomes (50 μg/ml) [43, 62]. After 7-day treatment, the cells were used for qRT-PCR, Western blot, and ALP staining. After 14-day treatment, these cells were used for alizarin red staining.

### Drugs treatment of cells

For exosomes inhibitor treatment, BMDMs were treated with GW4869 (#HY-19363, MCE, USA, 10 μM) [32, 63] after being mechanically stimulated. The same volume of DMSO was added as that for the controls. Two days later, the supernatants were collected for preparing conditioned medium.

For UCHL3 inhibitor treatment, BMSCs were treated with TCID (#HY-18638, MCE, USA, 10 μM) [35]. The same volume of DMSO was added as that for the controls. After being treated for 7 days, the cells were used for qRT-PCR, Western blot, and ALP staining. After being treated for 14 days, the cells were used for alizarin red staining.

### Lentivirus construction and infection

Lentivirus were constructed and infected as previously [64]. Briefly, overexpression lentiviruses of SMAD1 and their vector control (ov-Smad1, ov-NC) were purchased from Genechem (Shanghai, China). Lentiviruses and polybrene (5 μg/ml, Sigma) were added to the medium and incubated with BMSCs for 24 h at a multiplicity of infection (MOI) of 50. After 24 h, the medium was refreshed and the cells were used for the following experiments.

### CCK‑8 (Cell counting kit‑8) assay

After mechanical stimulation, BMDMs were incubated with 2 ml fresh medium and 200 μl CCK-8 reagent (#96,992, Sigma-Aldrich, USA) under 37 °C for 30 min, and then the absorbance was measured at 450 nm.

### Exosome purification, characterization and uptake

Exosomes were isolated and purified from macrophage culture supernatants following previously established protocol [65, 66]. Briefly, after culture for 48 h, the culture supernatants were collected and centrifuged at 300 g for 10 min, 2000 g for 10 min, and 10,000 g for 30 min to eliminate cells and debris. Subsequently, the supernatants were ultracentrifuged at 100,000 g for 70 min for two times, and the pellets were resuspended in PBS and kept under -80℃ (Additional file 1: Fig. S15).

Transmission electron microscopy (TEM) (JEM, Japan) and Western blot analysis were applied to assess the morphology and surface markers expression of exosomes. Exosomes concentration and size distribution were determined using Nanoparticle tracking analysis (NTA) (ZetaView, Particle Metrix, Germany).

The uptake of exosomes by BMSCs was confirmed through labeling exosomes using Dil as previously described [67]. Briefly, the purified exosomes were labeled with the membrane-labeling dye Dil (#V22888, Thermo Fisher Scientific, USA) following the manufacturer’s instructions, and these labeled exosomes were re-suspended in sterile PBS. Then with ultracentrifugation at 100,000 g for 70 min, the Dil-labeled exosomes were isolated. Next, Dil-EXOs were co-cultured with BMSCs for 12 h, after which these cells were fixed with 4% paraformaldehyde and photographed using fluorescence microscope (Leica Microsystems, Germany).

### RNA preparation and quantitative real-time PCR (qRT‒PCR)

In accordance with the operation instruction, the total RNA within the cells was isolated by RNA Isolation Kits (Omega, Guangzhou, China), and cDNA was reversely transcribed with the application of HiScript II Q RT SuperMix (Vazyme, China). Next, qRT-PCR was conducted by AceQ qPCR SYBR Green Master Mix (Vazyme, China) on the ABI QuantStudio7 real-time PCR system (Applied Biosystems, USA), and all the primer sequences were given in Table 1. Finally, all data were normalized to Gapdh expression and quantified with the 2^−ΔΔCT^ method.

**Table 1:** The sequences of the primers

qRT‒PCR primer name	Primer sequence (5′-3′)
Gapdh (forward)	TCCATGACAACTTTGGTATCG
Gapdh (reserve)	TGTAGCCAAATTCGTTGTCA
Alp (forward)	ATAACGAGATGCCACCAGAGG
Alp (reserve)	TTCCACATCAGTTCTGTTCTTCG
Runx2 (forward)	AGAATGGACGTGCCCCCTA
Runx2 (reserve)	CTGGGGAAGCAGCAACACTA
Col1 (forward)	CTGACTGGAAGAGCGGAGAG
Col1 (reserve)	CGGCTGAGTAGGGAACACAC
Ocn (forward)	TTCTGCTCACTCTGCTGACCC
Ocn (reserve)	CTGATAGCTCGTCACAAGCAGG
Uchl3 (forward)	AGCAATGCCTGTGGAACGAT
Uchl3 (reserve)	TTTGGCTCTCTCTTCAGGGC
Smad1 (forward)	ACCCCTACCACTATAAGCGAG
Smad1 (reserve)	TGCTGGAAAGAGTCTGGGAAC

### Western blotting

Western blotting was carried out as described previously [68]. Firstly, the total proteins in cell lysates were harvested, and then they were separated using SDS-PAGE gels. Later, the proteins that had been separated were transferred to polyvinylidene difluoride membranes, which were then blocked in the fat free milk (concentration of 5%). Subsequently, the following antibodies were incubated under 4 °C overnight: anti-RUNX2 (#12556, Cell Signaling Technology, 1:1000), anti-TSG101 (#ab125011, Abcam, 1:1000), anti-CD63 (#ab217345, Abcam, 1:1000), anti-Calnexin (#ab213243, Abcam, 1:1000), anti-UCHL3 (#D25E6, Cell Signaling Technology, 1:1000), anti-SMAD1 (#AP20642c, Abcepta, 1:1000), and anti-β-ACTIN (#4976, Cell Signaling Technology, 1:1000). After incubated with horseradish peroxidase‐conjugated secondary antibodies (Shengxing Biological, Nanjing, China) for 1 h, the specific bands were visualized with the enhanced chemiluminescence (Tanon). The relative density was measured using ImageJ software (V1.8.0, National Institutes of Health, USA).

### ALP staining

ALP staining was carried out using the 1-step NBT/BCIP reagent (#34042, Thermo Fisher Scientific, USA) after BMSCs were cultured for 7 days. Briefly, the cells were washed for two times by PBS, and then they were fixed in 4% paraformaldehyde for 30 min. After that, BMSCs were stained using BCIP/NBT substrate for 10 min and washed with PBS.

### Alizarin red staining

To detect matrix mineralization, alizarin red staining was conducted after BMSCs were cultured for 14 days. Later BMSCs was washed for two times by PBS and fixed in 4% paraformaldehyde for 30 min. Finally, they were stained using 2% alizarin red (#DS0072, Leagene, China) for 10 min and washed with deionized water.

### Cell immunofluorescent staining

BMSCs were fixed with 4% paraformaldehyde for 30 min and permeabilized with 0.5% Triton X-100 for 20 min. Then they were treated with goat serum for 30 min and incubated with the following primary antibodies overnight at 4 °C: anti-UCHL3 (#D25E6, Cell Signaling Technology, 1:200), anti-SMAD1 (sc-7965, Santa Cruz, 1:200). Subsequently, the species-matched secondary antibodies were used, and the nucleus were stained with DAPI.

### Coimmunoprecipitation (Co-IP) assay

Cells were harvested and lysed in IP buffer. Then, 50 μl protein-G agarose beads (#sc-2003, Santa Cruz) were added to 1 mg total protein, and incubated with rocking at 4 °C for 15 min. After centrifugation at 12,000 rpm for 10 min, the supernatant was collected and incubated with 2 μg anti-UCHL3 (#D25E6, Cell Signaling Technology), anti-SMAD1 (#AP20642c, Abcepta) or IgG (#A7028, Bytotime) antibodies at 4 °C for 2 h. Then 60 μl protein-G agarose beads were added, and the mixture was incubated with rocking at 4 °C overnight. After the agarose beads were collected, washed, and resuspended in 60 μl of 2% protein loading buffer, the samples were boiled for 10 min and used for Western blotting.

### Protein–protein docking

Protein–protein docking was performed using Alphafold2. The full-length sequence of protein was downloaded from UniProt (https://www.uniprot.org/). Then the two protein sequences were input into Alphafold2 (https://github.com/sokrypton/ColabFold) for docking.

### Proteomic analysis

Exosomes were isolated from BMDMs and MS-BMDMs supernatants. The protein extracted from exosomes was used for proteomic analysis by HOOGEN BIOTECH (Shanghai, China). In brief, the extracted protein from exosomes was subjected to reduction, alkylation and trypsin digestion, and then the digested protein was used for LC‒MS/MS analyses (Thermo Fisher Scientific, USA). After LC‒MS/MS, protein identification and quantitative analysis, as well as differential protein screening with a cut-off of fold change > 2.0 or < 0.5 and *P* values < 0.05, were performed. Then, bioinformatics analysis was conducted, and Gene Ontology and hierarchical clusters with corrected *P* values < 0.05 were considered significant.

### Statistical analysis

The data were expressed in the form of mean ± SD. We carried out statistical analysis using GraphPad Prism 8 software (GraphPad, USA). Two-tailed unpaired Student’s t-test were used for comparisons between 2 groups. And one-way ANOVA followed by Tukey's post-hoc multiple comparisons was conducted to comparing among 3 or more groups. *P* values < 0.05 were considered statistically significant.

## Supplementary Information

**Additional file 1: Fig. S1** Identification of BMSCs. **a** Osteogenic differentiation capability of third‐generation BMSCs detected by Alizarin red staining. Scale bar: 100 μm. **b** Adipogenic differentiation capability of third‐generation BMSCs detected by Oil red staining. Scale bar: 100 μm. **c** Chondrogenic differentiation capability of third‐generation BMSCs detected by Toluidine blue staining. Scale bar: 100 μm. **Fig. S2 a** Cell viability of BMDMs under the influences of different strain levels examined by CCK-8 assay (n = 3). **b** Cell viability of BMDMs under the influences of different strain durations examined by CCK-8 assay (n = 3). Data are shown as the mean ± SD. One-way ANOVA followed by Tukey’s post hoc multiple comparisons was performed. *P < 0.05, **P < 0.01; ns, not significant. **Fig. S3** Experimental design. The OTM model was generated in 2-month-old mice and exosome level was blocked by intraperitoneal injection of GW4869 every 2 days during loading. After 14 days of OTM, the maxillary was harvested. **Fig. S4, S6, S10, S12 a** Representative ALP staining images of the interradicular region of the first molar at day 14 after OTM treatment. The square frame represents the alveolar bone on the tension side of the first molar D: dentin. Scale bar: 200 μm. **b** Quantification from the square frame of a. ALP-positive surface relative to bone surface (%) on the tension side of the first molar (n = 6). Data are shown as the mean ± SD. One-way ANOVA followed by Tukey’s post hoc multiple comparisons was performed. *P < 0.05, **P < 0.01, ***P < 0.001; ns, not significant. **Fig. S5** Experimental design. The OTM model was generated in 2-month-old mice. The PBS, BMDM-EXOs, and MS-BMDM-EXOs were locally injected into the palatal gingiva of the loaded first molar every 3 days. After 14 days of OTM, the maxillary was harvested. **Fig. S7** Western blotting of UCHL3 protein level in BMSCs after treatment with the PBS, BMDM-EXOs and MS-BMDM-EXOs. **Fig. S8** The rule of finding the key functional protein in this study. **Fig. S9** Experimental design. The OTM model was generated in 2-month-old mice and UCHL3 level was inhibited by intraperitoneal injection of TCID every 2 days during loading. After 14 days of OTM, the maxillary was harvested. **Fig. S11** Experimental design. The OTM model was generated in 2-month-old mice. The PBS, MS-BMDM_siCon_-EXOs, and MS-BMDM_siUchl3_-EXOs were locally injected into the palatal gingiva of the loaded first molar every 3 days. After 14 days of OTM, the maxillary was harvested. **Fig. S13 a** qRT‒PCR of the mRNA expression of Smad1 in BMSCs after treatment with the lentiviruses of Ov-NC and Ov-Smad1 (n = 3). **b** Western blotting of SMAD1 protein level in BMSCs after treatment with the lentiviruses of Ov-NC and Ov-Smad1. **Fig. S14 a** Representative immunohistochemical staining of SMAD1 in the loaded alveolar bone at 14 d after OTM treatment (n = 6). D: dentin, Scale bar: 200 μm. **b** Quantification from a. SMAD1-positive surface relative to bone surface (%) in the loaded alveolar bone. Data are shown as the mean ± SD. One-way ANOVA followed by Tukey’s post hoc multiple comparisons was performed. ***P < 0.001; ns, not significant. **Fig. S15** The procedures of isolating and purifying the exosomes.

## Data Availability

The datasets used and analysed during the current study are available from the corresponding author on reasonable request.

## Abbreviations

OTM: Orthodontic tooth movement
BMSCs: Bone marrow-derived mesenchymal stem cells
MS: Mechanical stimulation
BMDMs: Bone marrow-derived macrophages
MS-BMDM-EXOs: Exosomes derived from mechanically stimulated BMDMs
UCHL3: Ubiquitin carboxyl-terminal hydrolase isozyme L3
SMAD1: Mothers against decapentaplegic homolog 1
qRT-PCR: Quantitative reverse transcription-polymerase chain reaction
ALP: Alkaline phosphatase
TEM: Transmission electron microscopy
NTA: Nanoparticle tracking analysis
HE: Hematoxylin–eosin staining
GO: Gene Ontology
MS-BMDM_siUchl3_-EXOs: Exosomes derived from BMDMs which were transfected with siUchl3 after being mechanically stimulated
Co-IP: Co-Immunoprecipitation
SD: Standard deviation
