# Supplementary material for: Mechanical force induces macrophage-derived exosomal UCHL3 promoting bone marrow mesenchymal stem cell osteogenesis by targeting SMAD1
Source: J Nanobiotechnology. 2023 Mar 14;21:88. doi: 10.1186/s12951-023-01836-z (PMC10012474; doi:10.1186/s12951-023-01836-z)
Supplement: Supplementary file 1 — Additional file 1: Fig. S1 Identification of BMSCs. a Osteogenic differentiation capability of third‐generation BMSCs detected by Alizarin red staining. Scale bar: 100 μm. b Adipogenic differentiation capability of third‐generation BMSCs detected by Oil red staining. Scale bar: 100 μm. c Chondrogenic differentiation capability of third‐generation BMSCs detected by Toluidine blue staining. Scale bar: 100 μm. Fig. S2 a Cell viability of BMDMs under the influences of different strain levels examined by CCK-8 assay (n = 3). b Cell viability of BMDMs under the influences of different strain durations examined by CCK-8 assay (n = 3). Data are shown as the mean ± SD. One-way ANOVA followed by Tukey’s post hoc multiple comparisons was performed. *P < 0.05, **P < 0.01; ns, not significant. Fig. S3 Experimental design. The OTM model was generated in 2-month-old mice and exosome level was blocked by intraperitoneal injection of GW4869 every 2 days during loading. After 14 days of OTM, the maxillary was harvested. Fig. S4, S6, S10, S12 a Representative ALP staining images of the interradicular region of the first molar at day 14 after OTM treatment. The square frame represents the alveolar bone on the tension side of the first molar D: dentin. Scale bar: 200 μm. b Quantification from the square frame of a. ALP-positive surface relative to bone surface (%) on the tension side of the first molar (n = 6). Data are shown as the mean ± SD. One-way ANOVA followed by Tukey’s post hoc multiple comparisons was performed. *P < 0.05, **P < 0.01, ***P < 0.001; ns, not significant. Fig. S5 Experimental design. The OTM model was generated in 2-month-old mice. The PBS, BMDM-EXOs, and MS-BMDM-EXOs were locally injected into the palatal gingiva of the loaded first molar every 3 days. After 14 days of OTM, the maxillary was harvested. Fig. S7 Western blotting of UCHL3 protein level in BMSCs after treatment with the PBS, BMDM-EXOs and MS-BMDM-EXOs. Fig. S8 The rule of finding the key functional p [file 12951_2023_1836_MOESM1_ESM.docx]

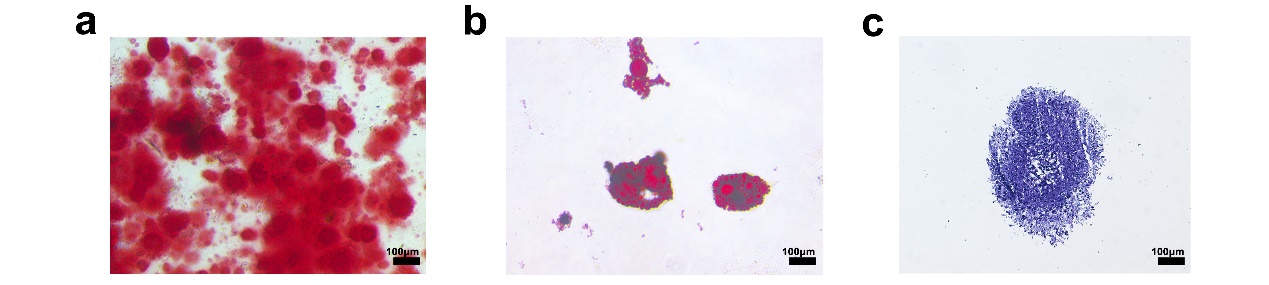


**Fig. S1** Identification of BMSCs. **a** Osteogenic differentiation capability of third‐generation BMSCs detected by Alizarin red staining. Scale bar: 100 μm. **b** Adipogenic differentiation capability of third‐generation BMSCs detected by Oil red staining. Scale bar: 100 μm. **c** Chondrogenic differentiation capability of third‐generation BMSCs detected by Toluidine blue staining. Scale bar: 100 μm.


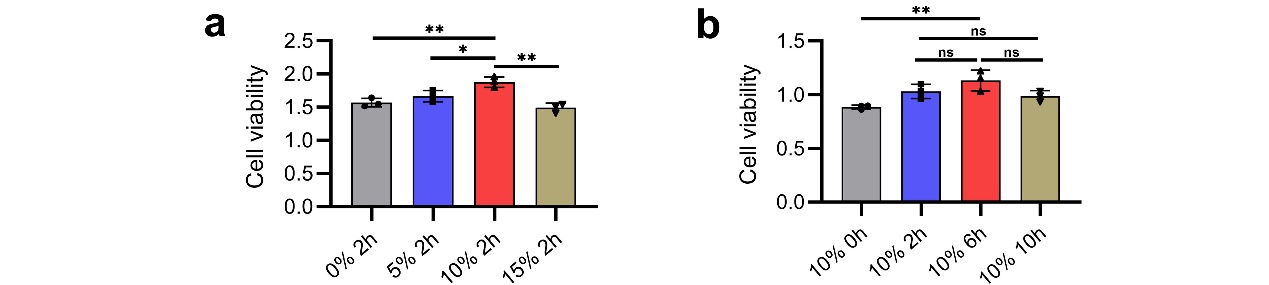


**Fig. S2 a** Cell viability of BMDMs under the influences of different strain levels examined by CCK-8 assay (n=3). **b** Cell viability of BMDMs under the influences of different strain durations examined by CCK-8 assay (n=3). Data are shown as the mean ± SD. One-way ANOVA followed by Tukey’s post hoc multiple comparisons was performed. **P* < 0.05, ***P* < 0.01; ns, not significant.


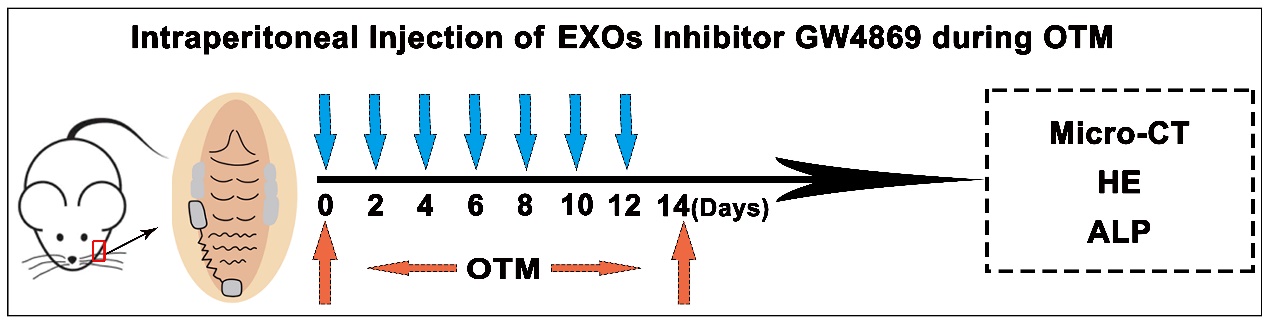


**Fig. S3** Experimental design. The OTM model was generated in 2-month-old mice and exosome level was blocked by intraperitoneal injection of GW4869 every 2 days during loading. After 14 days of OTM, the maxillary was harvested.


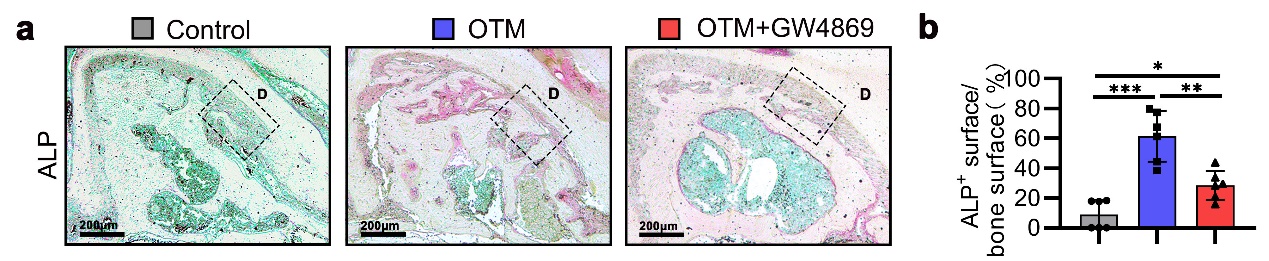


**Fig. S4** **a** Representative ALP staining images of the interradicular region of the first molar at day 14 after OTM treatment. The square frame represents the alveolar bone on the tension side of the first molar D: dentin. Scale bar: 200 μm. **b** Quantification from the square frame of a. ALP-positive surface relative to bone surface (%) on the tension side of the first molar (n=6). Data are shown as the mean ± SD. One-way ANOVA followed by Tukey’s post hoc multiple comparisons was performed. **P* < 0.05, ***P* < 0.01, ****P* < 0.001.


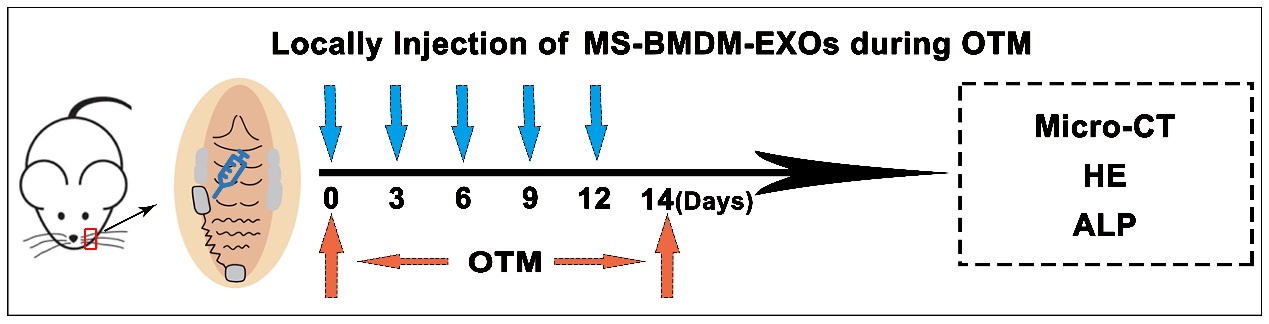


**Fig. S5** Experimental design. The OTM model was generated in 2-month-old mice. The PBS, BMDM-EXOs, and MS-BMDM-EXOs were locally injected into the palatal gingiva of the loaded first molar every 3 days. After 14 days of OTM, the maxillary was harvested.


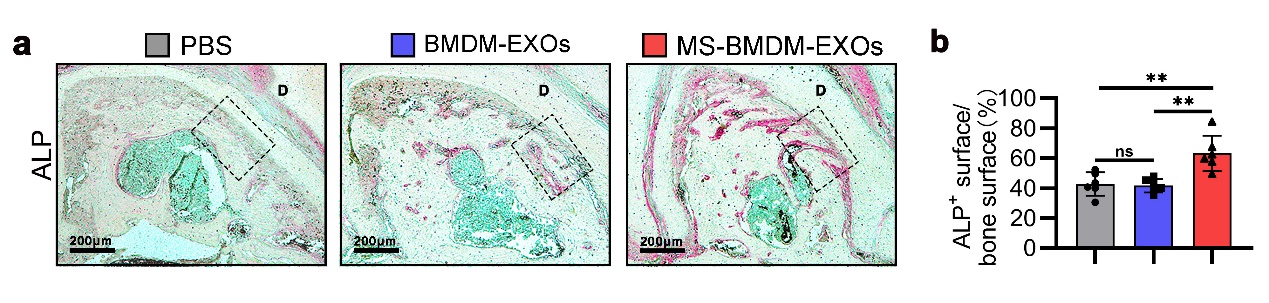


**Fig. S6** **a** Representative ALP staining images of the interradicular region of the first molar at day 14 after OTM treatment. The square frame represents the alveolar bone on the tension side of the first molar D: dentin. Scale bar: 200 μm. **b** Quantification from the square frame of a. ALP-positive surface relative to bone surface (%) on the tension side of the first molar (n=6). Data are shown as the mean ± SD. One-way ANOVA followed by Tukey’s post hoc multiple comparisons was performed. ***P* < 0.01; ns, not
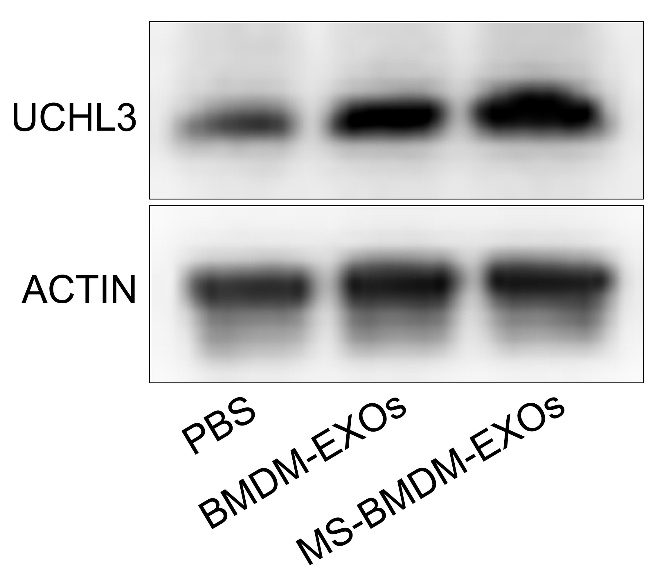
significant.

**Fig. S7** Western blotting of UCHL3 protein level in BMSCs after treatment with the PBS, BMDM-EXOs and MS-BMDM-EXOs.


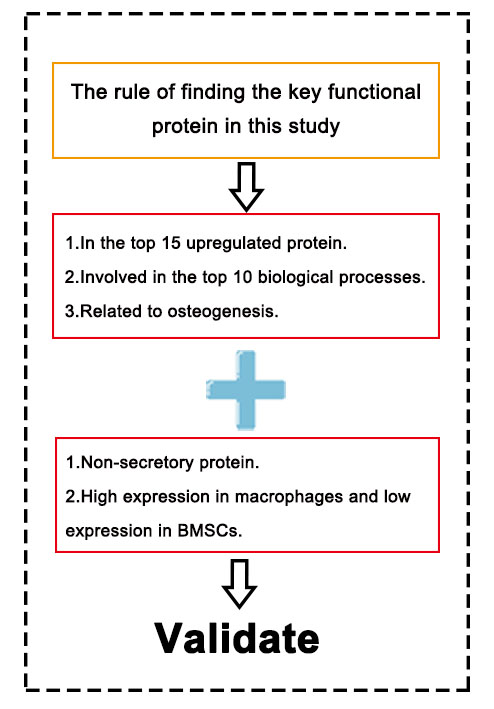


**Fig. S8** The rule of finding the key functional protein in this study.


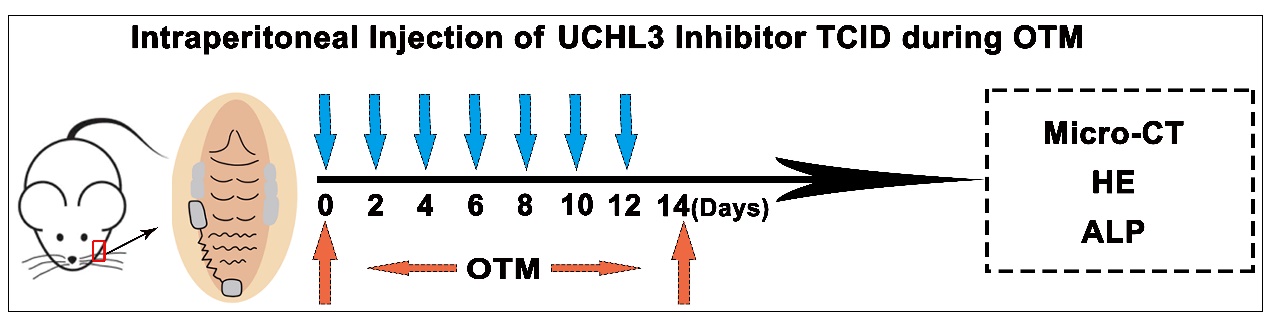


**Fig. S9** Experimental design. The OTM model was generated in 2-month-old mice and UCHL3 level was inhibited by intraperitoneal injection of TCID every 2 days during loading. After 14 days of OTM, the maxillary was harvested.


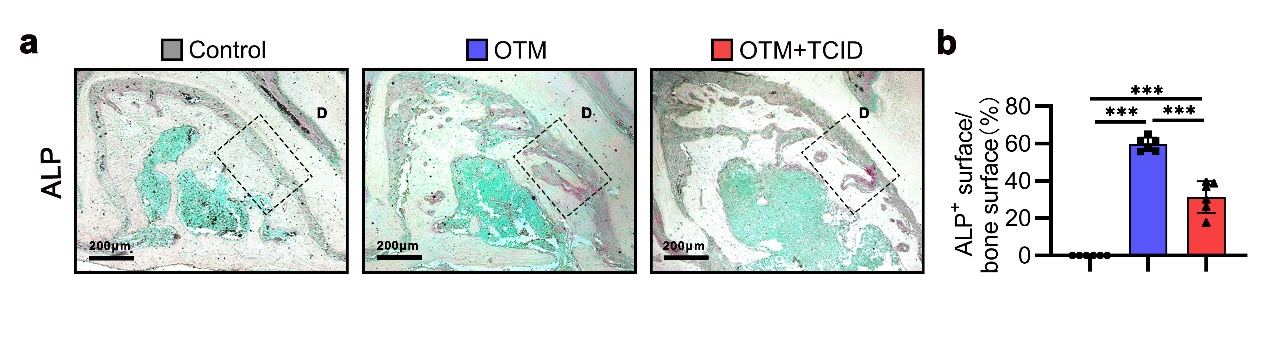
**Fig. S10** **a** Representative ALP staining images of the interradicular region of the first molar at day 14 after OTM treatment. The square frame represents the alveolar bone on the tension side of the first molar D: dentin. Scale bar: 200 μm. **b** Quantification from the square frame of a. ALP-positive surface relative to bone surface (%) on the tension side of the first molar (n=6). Data are shown as the mean ± SD. One-way ANOVA followed by Tukey’s post hoc multiple comparisons was performed. ****P* < 0.001.


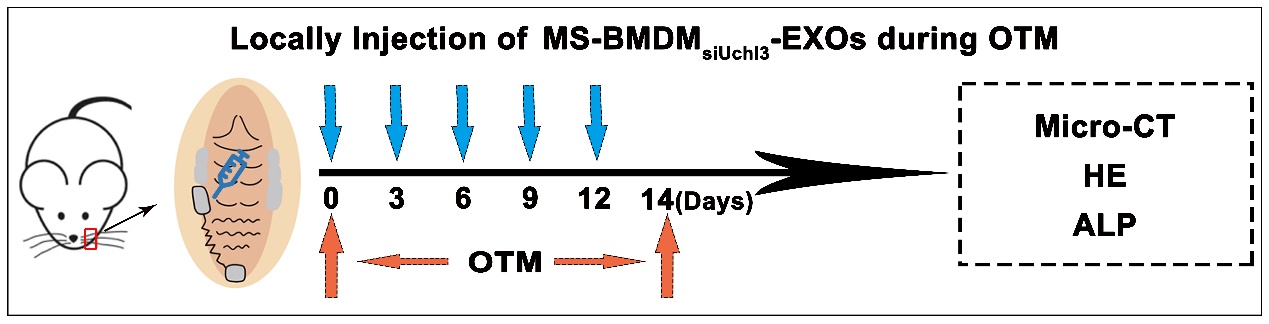


**Fig. S11** Experimental design. The OTM model was generated in 2-month-old mice. The PBS, MS-BMDM_siCon_-EXOs, and MS-BMDM_siUchl3_-EXOs were locally injected into the palatal gingiva of the loaded first molar every 3 days. After 14 days of OTM, the maxillary was harvested.


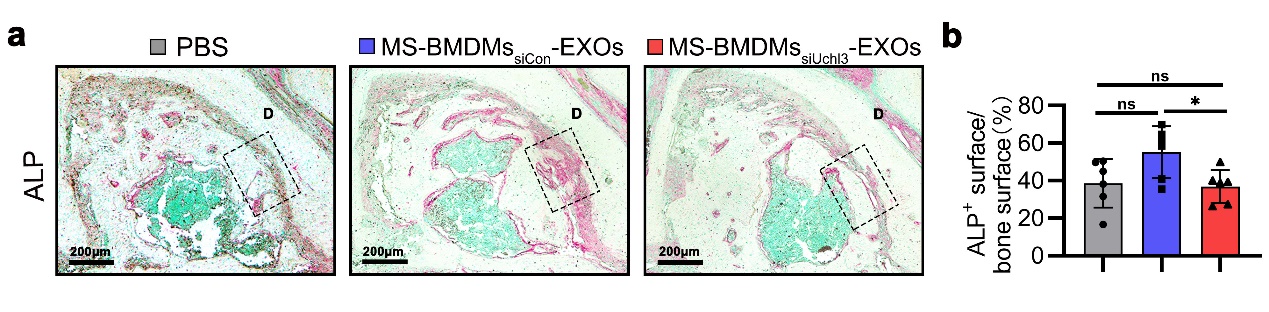


**Fig. S12** **a** Representative ALP staining images of the interradicular region of the first molar at day 14 after OTM treatment. The square frame represents the alveolar bone on the tension side of the first molar D: dentin. Scale bar: 200 μm. **b** Quantification from the square frame of a. ALP-positive surface relative to bone surface (%) on the tension side of the first molar (n=6). Data are shown as the mean ± SD. One-way ANOVA followed by Tukey’s post hoc multiple comparisons was performed. **P* < 0.05; ns, not significant.


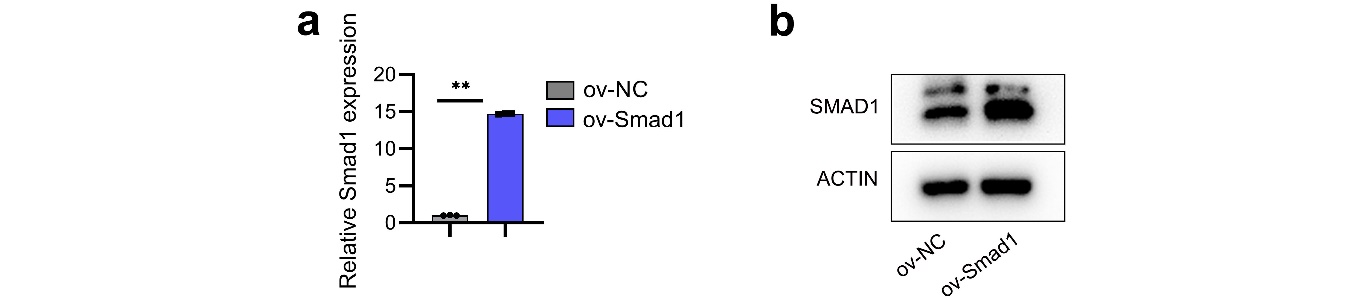


**Fig. S13 a** qRT‒PCR of the mRNA expression of Smad1 in BMSCs after treatment with the lentiviruses of Ov-NC and Ov-Smad1 (n=3). **b** Western blotting of SMAD1 protein level in BMSCs after treatment with the lentiviruses of Ov-NC and Ov-Smad1.


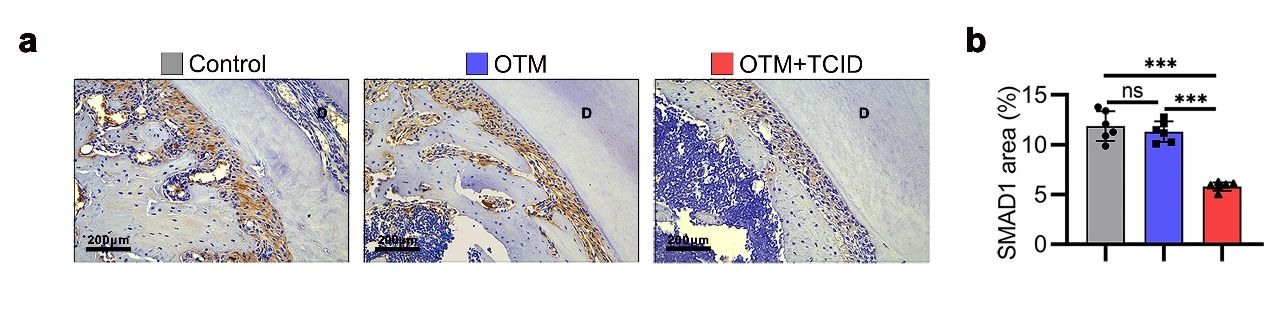


**Fig. S14** **a** Representative immunohistochemical staining of SMAD1 in the loaded alveolar bone at 14 d after OTM treatment (n=6). D: dentin, Scale bar: 200 μm. **b** Quantification from a. SMAD1-positive surface relative to bone surface (%) in the loaded alveolar bone. Data are shown as the mean ± SD. One-way ANOVA followed by Tukey’s post hoc multiple comparisons was performed. ****P* < 0.001; ns, not significant.


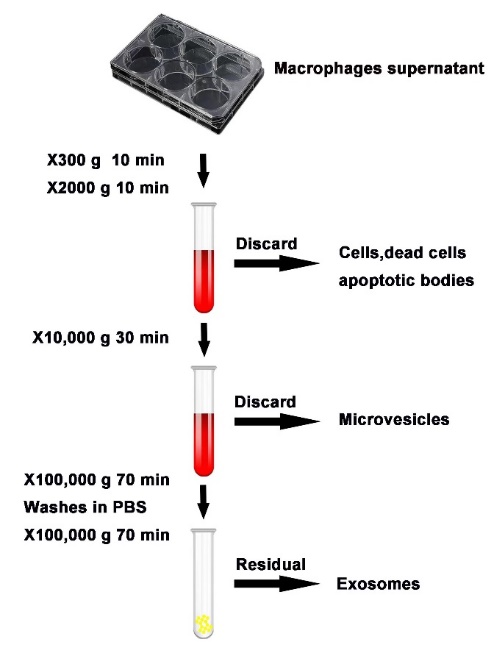


**Fig. S15** The procedures of isolating and purifying the exosomes.
